# Dated phylogeny and dispersal history of the butterfly subfamily Nymphalinae (Lepidoptera: Nymphalidae)

**DOI:** 10.1038/s41598-017-08993-w

**Published:** 2017-08-18

**Authors:** Chengyong Su, Qinghui Shi, Xiaoyan Sun, Junye Ma, Chunxiang Li, Jiasheng Hao, Qun Yang

**Affiliations:** 1 College of Life Sciences, Anhui Normal University, Wuhu, 241000 China; 2 Nanjing Institute of Geology and Paleontology, Chinese Academy of Sciences, Nanjing, 210008 China; 3 Xuzhou College of industrial technology, Xuzhou, 221140 China

## Abstract

The origin and dispersal history of the large butterfly subfamily Nymphalinae are not fully understood, due to internal phylogenetic and time calibration issues. We conducted phylogenetic and dating analyses using mitochondrial and nuclear genes of biogeographically diverse groups of the Nymphalinae in order to resolve some controversial relationships and the paleobiogeographic pattern of the subfamily. Our results support the sister relationship of *Vanessa* (Tribe Nymphalini) and the *Nymphalis*-group, and the grouping of the three old-world genera (*Rhinopalpa*, *Kallimoides* and *Vanessula*) within Tribe Victorinini. Molecular dating analyses invoking two additional calibrations under the butterfly-host plant coevolutionary scenarios result in a relatively deeper divergence of the subfamily’s two major clades (Nymphalini and the Kallimoids), compatible with the Cretaceous floral turnover scenario during the so-called Cretaceous Terrestrial Revolution. Phylobiogeographic analyses reveal that the Oriental region is probably the center of early divergences for Nymphalinae after the Cretaceous-Paleogene (K-Pg) mass extinction, followed by repeated dispersals into the rest of the Old World and the New World during various periods beginning in Eocene. The biogeographic history indicates that temperature changes and host-plant diversification may have facilitated the dispersals of this butterfly subfamily, with accelerated global colonization during the middle to late Miocene.

## Introduction

The subfamily Nymphalinae consists of the tribes Nymphalini, Melitaeini, Kallimini, Victorinini, Junoniini, and probably the Coeini^1^. Previous studies, based on various gene sequences of taxa with several missing genera, resulted in weakly supported and unstable trees that need clarification^1–3^. For example, the clade comprising the genera *Vanessa* and *Hypanartia* was unstable; the basal relationships of the kallimoid clade and the sister relationship of Kallimini and Melitaeini were poorly resolved; the phylogenetic positions of the three monotypic genera (*Kallimoides*, *Rhinopalpa* and *Vanessula*) were also uncertain.

Within Nymphalinae, the tree topology and molecular dating results may be significantly affected by taxon sampling, time-calibration points and parameter models^2–5^. The ages of diversification of major lineages and the influenced historical biogeography of this butterfly subfamily were still fraught with uncertainties, due to computational constraints, limited exemplar species, scarce and relatively young fossils, and potentially underestimated ages of host plants as maximum prior time constraints in previous studies^1–3^. Butterflies and their host plants are often found to coevolve and phylogenetically conserved^6–10^. Previous studies indicate that butterfly lineages with inferred major historical host shifts showed significant diversification accelerations. Some key plant groups (e.g., Acanthaceae, Asteraceae, Brassicales) appear to have been used by butterfly as larval hosts in close evolutionary time to the appearance of the host plants^10–13^. Therefore, we consider that the origins and divergences of the butterflies’ host plants, as well as the fossil records of butterflies, are relevant and significant to the dating of butterfly divergences and their biogeographical inference.

In this study, building on the pioneering work of Wahlberg and colleagues, we rely on comprehensive sampling of Nymphalinae butterflies, comprising 32 new sequenced specimens in China, to conduct multiple phylogenetic analyses using mitochondrial and nuclear gene sequences; the new phylogeny and multiple time calibrations, including secondary calibrations from relevant host plants with new strategies, were then utilized to estimate the divergence times. We also reconstructed the ancestral geographic distribution and the historical processes leading to the current worldwide biogeographical pattern of this butterfly group, with a combination of different biogeographic analysis.

## Results

### Phylogeny

**Figure 1:**
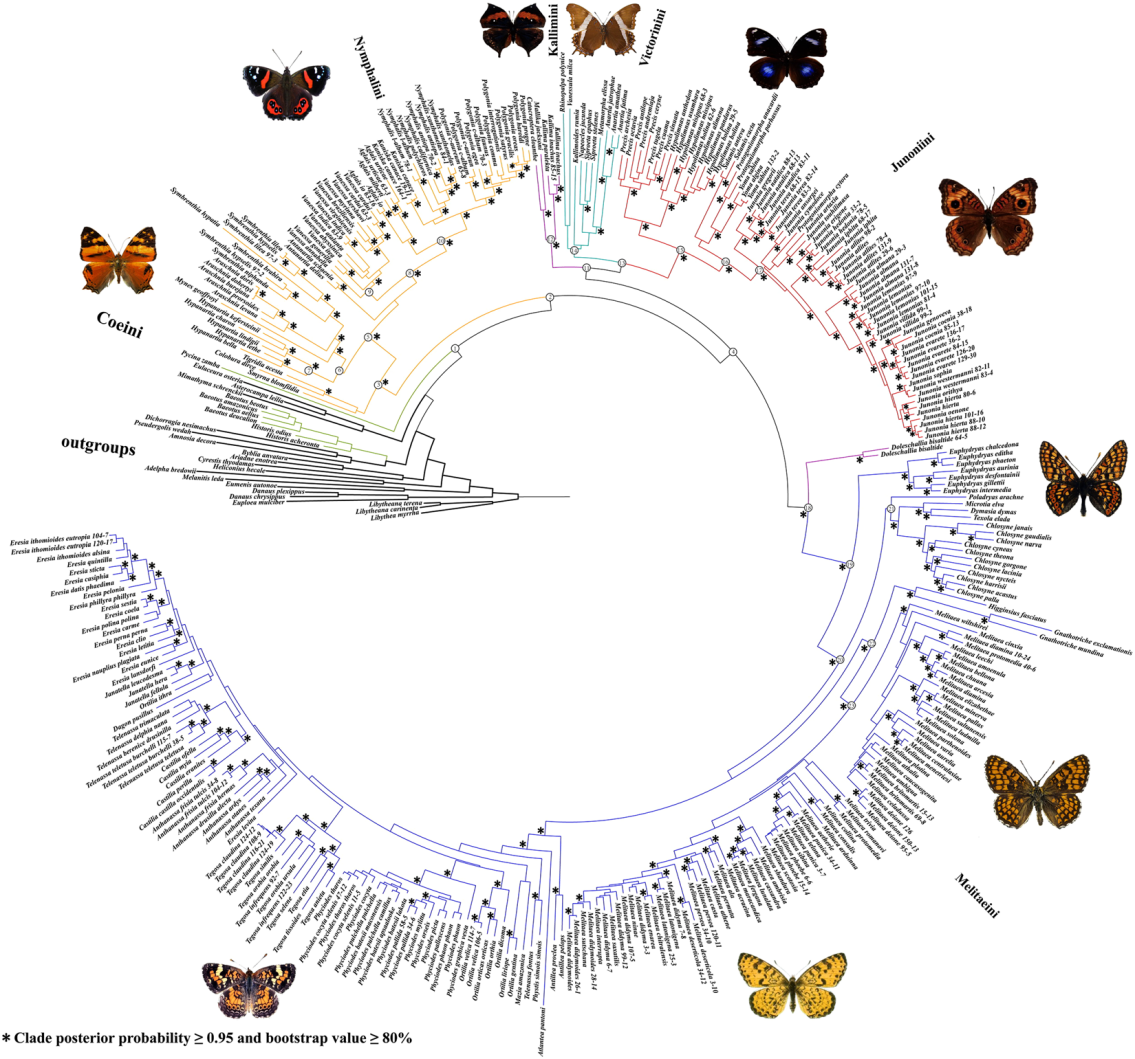
The specimen-level phylogenetic relationships of Nymphalinae based on maximum likelihood and Bayesian analyses (see under Methods). Images of *Hypanartia lethe* and *Vanessa gonerilla* from https://en.wikipedia.org/wiki/File:Hypanartia_lethe_dorsal.jpg and https://commons.wikimedia.org/wiki/File:NZ_Red_Admiral_(Vanessa_gonerilla)-2_edit.jpg, respectively, following the same license terms (https://creativecommons.org/licenses/by-sa/4.0/legalcode). Other images made by Chengyong Su.

We assembled the dataset with sampling the largest number of species and genera of this subfamily so far (comprising one mitochondrial and two nuclear gene sequences) to conduct phylogenetic inferences. Both ML and BI trees presented here are extremely similar in their topologies (Fig. 1 for specimen-level tree and Supplementary Fig. S1 for species-level tree; nodes with asterisks were stable in all analyses), and generally support previous phylogenetic hypotheses of the Nymphalinae^1, 3, 14^. However, our results were different from previous analyses in the some crucial aspects.

*Historis* + *Baeotus* of the tribe Coeini are far away from the rest of Nymphalinae, which was largely in line with previous analyses by Wahlberg *et al*.^1^, whereas significantly different from their subsequent study^3^. *Pycina*, morphologically intermediate between *Historis/Baeotus* and *Colobura* in larvae and pupae^15^, was recovered to be sister of the other Nymphalinae (PP = 0.95; BS = 59%).

Within the monophyletic Nymphalini clade, contrary to the results of earlier studies^1, 3^, the position of *Vanessa* (node 9, Fig. 1) is well-supported (PP = 1.00; BS = 89%) as the sister to the *Nymphalis*-group (node 10, containing *Aglais*, *Kaniska*, *Nymphalis* and *Polygonia*), which was also supported by two unique synapomorphies from the morphological, ecological, and behavioral analyses^14, 16^. More importantly, the genus *Hypanartia* (node 7) forms one of the basal genera in Nymphalini, sister to the monophyletic group formed by *Araschnia*, *Symbrenthia* and *Mynes*, most of which have the same ancestral states of no discal spot and only very slight pupal anterior projections^14^. Moreover, our results suggest that *Antanartia* is sister to the group of *Nymphalis*-group + *Vanessa* (node 8) with weak support, which seems concordant with shared morphological characters of the pupal anterior projections, the presence and shape of the discal spot^14^. As for the Kallimoid clade (node 4), our ML and BI analyses recovered the monophyly of Melitaeini, Junoniini, and Victorinini containing three Old World (OW) monotypic genera (*Rhinopalpa*, *Kallimoides* and *Vanessula*) and four New World (NW) genera (*Napeocles*, *Siproeta*, *Metamorpha* and *Anartia*) with high posterior probability (PP = 0.99). Because *Doleschallia* is apparently sister to the Melitaeini (PP = 0.99; BS = 85%), our results do not support the Kallimini of Wahlberg *et al*.^1^ that included *Doleschallia*, as found in a previous study^3^. The remaining members of the Kallimini (node 12, containing *Catacroptera*, *Kallima* and *Mallika*) were recovered as a sister group of Victorinini + Junoniini, not the Melitaeini; it is noted that a few basal nodes are weakly supported here.

The species-level MP tree is mostly concordant with the ML and BI trees, especially of the position of *Hypanartia* and *Vanessa*. The major differences are those about the position of Kallimini and Victorinini clades, which seems to be sister to the remainder of the Kallimoid clade, as found in a previous study^3^. *Antanartia* is not always found to be sister to the clade of *Vanessa* + *Nymphalis*-group here, but is a basal basal clade in the Old Nymphalini (node 5, Nymphalini clade excluding three Neotropical genera *Smyrna*, *Colobura* and *Tigridia*)^1, 2^.

### Divergence time estimates

**Figure 2:**
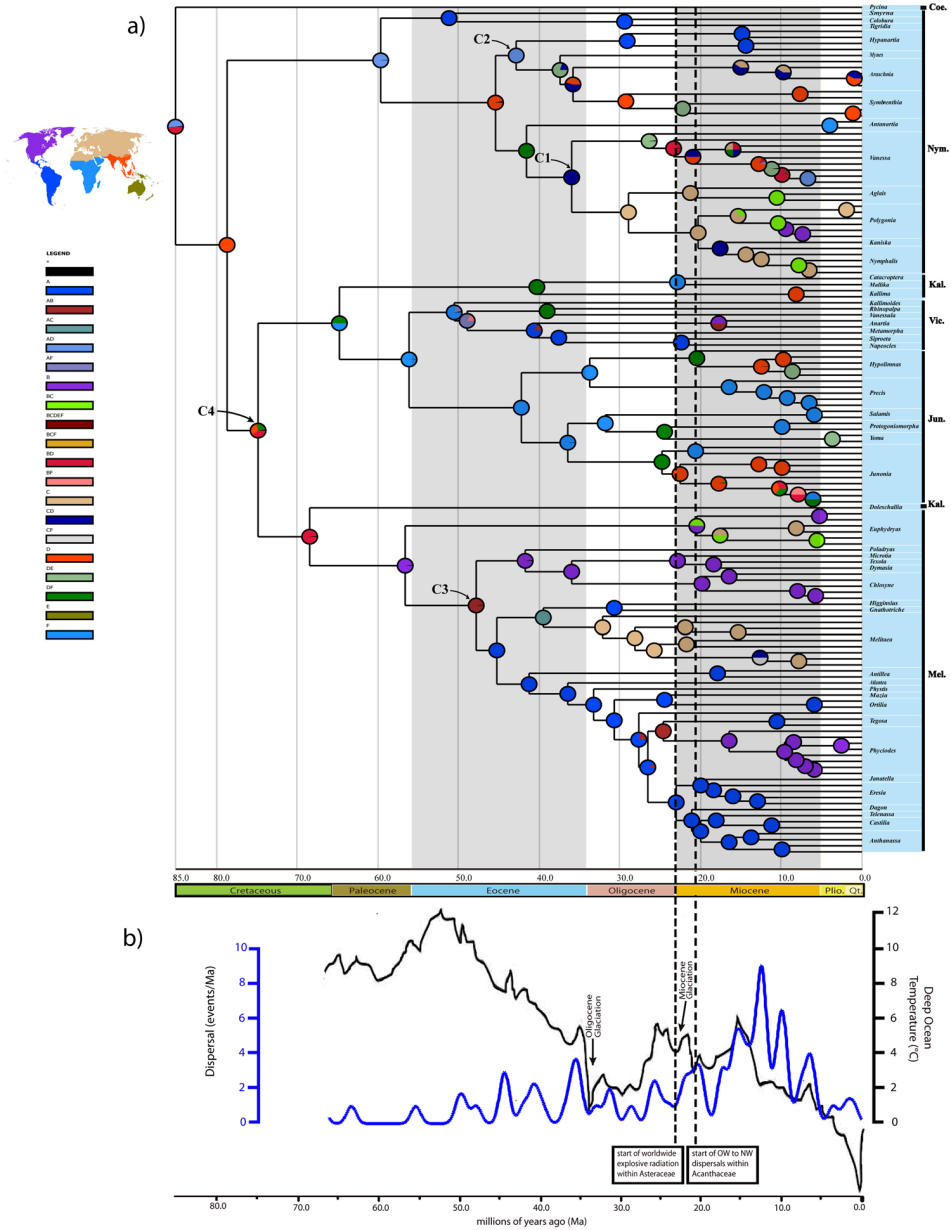
(**a**) Estimated time tree of the Nymphalinae (under Calibration Plan 1 A) with the results of the ancestral area reconstruction from S-DIVA analysis; C1, C2, C3, and C4 show the time calibration points (see under Methods); the map of biogeographic realms freely from https://commons.wikimedia.org/wiki/File:Ecozones.svg, following the same license terms (https://creativecommons.org/licenses/by-sa/4.0/legalcode). Coe., Coeini; Nym., Nymphalini; Kal., Kallimini; Vic., Victorinini; Jun., Junoniini; Mel., Melitaeini; Biogeographical realms: A, Neotropical; B, Nearctic; C, Palaearctic; D, Oriental; E, Australasian; F, Afrotropical; (**b**) Climatic-dispersal curve for Nymphalinae derived from S-DIVA analysis. Reconstruction of deep ocean temperatures (black curve, as a proxy for global temperature) is derived from oxygen isotopes corrected for variation in global ice volume (from Working Group I, 2007 Intergovernmental Panel for Climate Change report; see Fig. 6.1 therein); the time-events curve (blue curve) shows the changes in frequency of dispersal. OW, Old World; NW, New World.

We found that under Calibration Plan 1 A (3 calibration points: C1 (37.2–33.9 Ma (million years ago) for the divergence time between *Vanessa* and its sister group), C3 (49–39 Ma for the divergence time between the *Chlosyne*-group and its sister clade), C4 (81.9–71.9 Ma for the crown age of the Kallimoid clade), Fig. 2a), the estimated clade ages (Table 1) average ca. 2.5 m.y. (million years) older than under Calibration Plan 2 A (3 calibration points: C2 (37.2–33.9 Ma for the divergence time between *Hypanartia* and its sister group), C3, C4), and ca. 3.5 m.y. older than under Calibration Plan 1B (2 calibration points: C1, C3), and average up to 7 m.y. older than under Calibration Plan 2B (2 calibration points: C2, C3). We obtained age estimates here with variations lower than those in a previous study^2^, most likely due to multiple calibrations adopted in this study. It is noted that enforcing a time prior of the Kallimoid clade (C4, Fig. 2a) yielded older estimates of clade ages in both Calibration strategies 1 and 2, regardless of other time priors (Table 1). For example, the age of crown Nymphalinae was 85.1 Ma in Calibration Plan 1 A versus 76.7 Ma in Calibration Plan 1B and 82.7 Ma in Calibration Plan 2 A versus 70.8 Ma in Calibration Plan 2B (see Table 1 for 95% highest posterior density (HPD) intervals). Calibration Plan 1 A, under which posterior age distributions closely approximate ages of the priors in alignment-free BEAST analysis, is preferred in this study because the butterfly-host plant coevolutionary scenarios is considered significant in butterfly phylogenetic history and the global rise of the host plants (e.g., Rosales, Lamiales and Asterales) at about 90–110 Ma^17–19^ is treated as secondary calibration that could potentially improve the dating procedures.

**Table 1:** Comparison of crown ages of major lineages and the most recent common ancestor (MRCA) of major splits in Nymphalinae across four different calibration stategies (1 A, 1B, 2 A, 2B; see under Methods).

Major lineage/split	Node	Calibration Plans			
		1 A	1B	2 A	2B
Nymphalinae	1	85.1[73.9–98.2]	76.7[63.5–91.7]	82.7[72.5–95.2]	70.8[58.7–84.1]
Nymphalini/Kallimoid clade	2	78.7[72.3–85.4]	70.5[61.2–80.9]	76.9[70.9–83.1]	65.2[56.6–73.5]
Nymphalini	3	59.7[50.7–69.3]	55.5[46.9–64.5]	54.4[45.7–64.0]	50.3[43.1–58.3]
Kallimoid clade	4	74.9[70.4–79.7]	66.3[57.4–75.2]	73.8[68.9–78.1]	61.6[53.5–69.2]
Old Nymphalini	5	45.4[39.5–51.3]	43.1[38.0–48.6]	39.7[36.1–44.1]	38.2[35.2–41.6]
*Hypanartia*/sister clade	6	42.9[37.1–49.1]	40.7[35.2–46.5]	36.6[34.6–38.6]	36.0[34.0–38.0]
*Hypanartia*	7	29.2[21.7–36.5]	27.4[20.2–34.5]	25.6[20.0–31.2]	24.6[18.9–30.4]
*Vanessa*/*Nymphalis*-group	8	35.9[34.0–38.1]	35.6[33.6–37.7]	32.8[28.2–37.7]	30.9[26.1–35.6]
*Vanessa*	9	26.4[21.0–31.9]	25.3[19.7–31.1]	24.4[19.1–29.0]	22.6[17.5–27.7]
*Nymphalis*-group	10	28.9[24.3–33.1]	28.1[23.0–32.5]	26.6[21.8–31.3]	24.8[20.1–29.6]
Victorinini + Junoniini/Kallimini	11	64.8[55.3–74.4]	58.8[49.1–69.3]	62.2[52.1–72.0]	54.3[45.3–63.3]
Kallimini	12	40.2[27.6–53.9]	37.0[25.0–49.4]	38.2[25.7–51.1]	34.1[23.0–45.5]
Victorinini/Junoniini	13	56.2[47.3–64.5]	51.4[42.7–60.1]	53.5[45.5–62.3]	47.4[39.6–55.6]
Victorinini	14	50.5[42.1–59.0]	46.3[37.6–54.9]	48.0[39.8–56.7]	42.6[34.8–50.6]
Junoniini	15	42.3[35.3–49.8]	39.1[32.2–46.4]	39.9[33.0–46.9]	36.1[29.6–42.7]
*Junonia*/sister clade	16	36.5[29.9–43.3]	33.8[27.3–40.6]	34.4[28.2–41.2]	31.2[25.4–37.5]
*Junonia*	17	24.7[19.3–30.1]	23.0[17.6–28.2]	23.3[18.4–28.8]	21.2[16.6–26.2]
Melitaeini/sister clade	18	68.5[62.5–74.7]	66.3[57.4–75.2]	67.3[61.1–73.5]	61.6[53.5–69.2]
Melitaeini	19	56.6[50.5–62.7]	52.5[46.1–59.1]	55.3[49.2–61.4]	49.5[43.8–55.5]
*Chlosyne*-group/sister clade	20	47.8[43.5–51.9]	45.3[40.7–49.7]	46.5[42.5–50.7]	43.2[39.2–47.7]
*Chlosyne*-group	21	41.7[35.0–48.0]	39.4[32.8–46.2]	40.4[33.8–46.9]	37.3[30.5–43.3]
*Melitaea*/sister clade	22	39.5[33.4–44.8]	37.3[31.4–43.1]	38.1[32.4–43.8]	35.3[29.6–41.0]
*Melitaea*	23	32.1[26.0–38.1]	30.2[24.0–36.0]	30.7[24.6–36.7]	28.2[22.4–34.0]

Notes: Numbers in brackets refer to 95% HPD intervals of clade ages (note that because HPD intervals are calculated from all trees, node ages occasionally fall outside the interval).

In our analysis under Calibration Plan 1 A, as shown in Fig. 2 and Table 1, the stem Nymphalini lineage branched off the clade comprising the rest of the Nymphalinae (the Kallimoid clade) about 78.7 Ma. The two major clades (Nymphalini and the Kallimoid clade) originated during Late Cretaceous and crossed the Cretaceous-Paleogene (K-Pg) boundary (ca. 66 Ma). The extant Nymphalini (node 3, Fig. 1) further diverged during late Paleocene (ca. 59.7 Ma), when the common Neotropical clade comprising *Smyrna*, *Colobura* and *Tigridia* diverged from the other clade of Nymphalini.

The time estimate of the Kallimoid clade (node 4, Fig. 1) (ca. 79–77 Ma under Calibration Plans 1 A and 2 A, Late Cretaceous) is much earlier than in previous studies (ca. 60–50 Ma)^2, 3^. Though the basal relationships of the Kallimoid clade were poorly supported, the stem Kallimini lineage branched off the clade Victorinini + Junoniini about 64.8 Ma (Paleocene), and the stem Melitaeini lineage originated during Late Cretaceous (Table 1). Our estimation under Calibration Plan 1 A resulted in subsequent divergences of the tribes mainly during Eocene, and almost all sampled genera diverged from sister genera before the middle Miocene (ca. 18 Ma). Relaxing the assumptions of the minimum time priors derived by the maximum minus 5, 15 and 20 m.y., respectively, under the modified Calibration Plan 1 A (see under Methods), did not have strong effect on the estimated divergence ages (see Supplementary Table S4). Though the dating results sometimes varied with different priors, the main patterns of the evolutionary time were roughly in a state of consistency (e.g., the Kallimoid clade diverged from the Nymphalini in Late Cretaceous, and both the stem groups crossed the Cretaceous-Paleogene (K-Pg) boundary).

Our divergence time estimates of the Nymphalinae (about 85.1 Ma under Calibration Plan 1 A) and the major subordinate tribes, as shown in Fig. 2, are coeval with the Cretaceous Terrestrial Revolution (KTR, 125 to 80 Ma), when angiosperms replacing ferns and gymnosperms rose from nearly 0 to 80% in fossil flora^20, 21^, and the global rise of the butterfly-host plants (e.g., Rosales, Lamiales and Asterales) at about 110–90 Ma^17–19^. These phylochronologic estimates, coincident with the paleofloral events, suggest that the origin and early divergence of the nymphaline butterflies were probably related with the Middle and Late Cretaceous rise of angiosperms, especially the butterfly-host plants; later divergences of the nymphaline subordinate tribes (ca. 65–40 Ma, Table 1), during Paleocene and early Eocene after the K-Pg mass extinction event, were likely linked with the warm climate and angiosperm recovery after the mass extinction. Detailed results of the divergence time estimates are shown in Table 1.

### Historical biogeography

Our results support the hypothesis by Wahlberg *et al*.^1^ that the most recent common ancestor of Nymphalinae was widely distributed prior to the K-Pg boundary ca. 66 Ma. However, the originating area of the Nymphalinae (excluding *Historis* and *Baeotus*) was unclear because of the insufficient outgroup sampling, and uncertainty of its sister group in previous studies^1, 3, 22^. Under the improved phylogenetic framework (see above) with the ancestral area reconstruction methods (S-DIVA, DEC and S-DEC analyses; see under Methods below), it is found that the clade Nymphalini (node 3, Fig. 1) probably diverged and survived in the Neotropical and Oriental regions (marginal probability 99% in S-DIVA analysis) (Fig. 2a; Table 2), largely in line with the previous study by Wahlberg *et al*.^3^. As the sister group of *Hypanartia*, the group comprising *Araschnia*, *Symbrenthia* and *Mynes* likely originated in the Oriental region earlier than 35 Ma, with its subsequent dispersal to the Palaearctic and Australasian regions. As the sister group of the worldwidely distributed genus *Vanessa*, the *Nymphalis*-group (node 10, Fig. 1) diverged ca. 28.9 Ma in the Palaearctic region with marginal probability of 100% in S-DIVA analysis. These results were largely congruent with one of previous hypotheses that ancestors of different lineages in Nymphalini could have spread in the Neotropical, Palaearctic and Afrotropical (e.g., the genus *Antanartia*) regions^1^. Our results also suggested that the Old Nymphalini clade (node 5, Fig. 1) had probably colonized the New World in Eocene from the Old World (where the genus *Hypanartia* originated as a related fossil *Prodryas persephone* was found in late Eocene Florissant Shale Lagerstatte in Colorado). However, the clade comprising *Smyrna*, *Colobura* and *Tigridia*, probably underwent a relatively longer period of independent evolution in the Neotropical region (Fig. 2a).

**Table 2:** Ancestral area reconstruction for major clades of Nymphalinae.

Major lineage/split	Node	Probability of ancestral areas			Direction	Temporal range
		DEC (Lagrange) ([area]RP)	S-DEC (Bays-Lagrange) ([area]RP)	S-DIVA ([area]MP)		
Nymphalinae	1	[AD]0.54 [AB]0.29	[AD]0.42 [AB]0.31 [AF]0.14	[AD]0.49 [BD]0.49	—	Middle to Late Cretaceous
Nymphalini/Kallimoid clade	2	[AD]0.50 [B]0.21 [AB]0.15	[AD]0.39 [B]0.20 [AB]0.16	[D]1.00	D → B(A)	Late Cretaceous
Nymphalini	3	[AD]0.38 [A]0.24 [B]0.18	[A]0.34 [AD]0.30 [B]0.17	[AD]0.99	V	Late Cretaceous-Palaeocene
Kallimoid clade	4	[D]0.49 [B]0.27 [BD]0.13	[D]0.32 [B]0.25 [BD]0.14	[BD]0.40 [D]0.32 [DF]0.26	D → B/F	Late Cretaceous
Old Nymphalini	5	[AD]0.52 [BD]0.36 [D]0.11	[AD]0.54 [BD]0.33	[D]0.97	D → A/F	Eocene
*Hypanartia*/sister clade	6	[AD]0.52 [BD]0.40	[AD]0.54 [BD]0.39	[AD]1.00	D → B(A)	Eocene
*Hypanartia*	7	[AB]1.00	[AB]0.86	[A]1.00	B → A	Late Eocene-Early Miocene
*Vanessa*/*Nymphalis*-group	8	[D]0.63 [CD]0.37	[D]0.57 [CD]0.38	[CD]1.00	D → Wordwide	Late Eocene
*Vanessa*	9	[DE]0.59 [D]0.26	[DE]0.53 [D]0.29	[DE]0.98	D → Wordwide	Oligocene-Early Miocene
*Nymphalis*-group	10	[CD]0.76 [C]0.24	[CD]0.69 [C]0.26	[C]1.00	C → D ﻿﻿﻿C → B(A)	Oligocene
Victorinini + Junoniini/Kallimini	11	[DF]0.44 [BF]0.27 [D]0.15	[DF]0.34 [BF]0.20 [D]0.20	[F]0.51 [DF]0.49	D → F	Late Cretaceous-Palaeocene
Kallimini	12	[DF]0.82 [F]0.18	[DF]0.81 [F]0.16	[DF]1.00	V	Eocene-Middle Oligocene
Victorinini/Junoniini	13	[F]0.46 [BF]0.31 [DF]0.17	[F]0.35 [BF]0.27 [DF]0.18	[F]0.99	F → B(A) F → D	Palaeocene-Middle Eocene
Victorinini	14	[BF]0.50 [F]0.37 [DF]0.12	[BF]0.40 [F]0.26 [AF]0.14	[F]0.80 [AF]0.18	F → B(A) F → D	Late Palaeocene-Middle Eocene
Junoniini	15	[F]0.77 [DF]0.23	[F]0.74 [DF]0.25	[F]1.00	F → D(E)	Eocene
*Junonia*/sister clade	16	[F]0.75 [DF]0.25	[F]0.75 [DF]0.25	[F]0.99	F → D	Late Eocene- Middle Oligocene
*Junonia*	17	[DF]1.00	[DF]0.90	[DF]1.00	F → D(E)	Oligocene-Early Miocene
					F/D → B(A)	
Melitaeini/sister clade	18	[BD]0.50 [B]0.26 [D]0.16	[BD]0.42 [B]0.25 [D]0.17	[BD]0.98	D → E D → C/B	Late Cretaceous-Palaeocene
Melitaeini	19	[B]0.83 [BC]0.17	[B]0.58 [BC]0.14	[B]0.98	B/C → A	Palaeocene-Early Eocene
*Chlosyne*-group/sister clade	20	[AB]1.00	[AB]0.80 [A]0.14	[AB]0.99	B → A	Early to Middle Eocene
*Chlosyne*-group	21	[AB]0.56 [B]0.44	[AB]0.52 [B]0.40	[B]0.98	B → A	Eocene
*Melitaea*/sister clade	22	[AC]0.86	[AC]0.82	[AC]1.00	V	Middle to Late Eocene
*Melitaea*	23	[C]0.84 [CD]0.16	[C]0.83 [CD]0.12	[C]1.00	C → D/F	Late Eocene-Oligocene

Node labels correspond to Table 1 and Fig. 1. Direction reflects the path of the dispersal. Temporal range considers the age value of major lineages or splits under Calibration Plan 1 A, as shown in Table 1. Abbreviations: S-DIVA, Statistical Dispersal-Vicariance Analysis; DEC (Lagrange), Dispersal-Extinction-Cladogenesis Analysis; S-DEC (Bays-Lagrange), Statistical DEC Analysis; RP, relative probability; MP, marginal probability; V, vicariance. Biogeographical areas: A, Neotropical; B, Nearctic; C, Palaearctic; D, Oriental; E, Australasian; F, Afrotropical. The slash (/) between different realms means the diverged dispersal route for one clade; the realm in the parentheses means the destination for some groups in the same route of dispersal.

The Kallimoid clade (node 4, Fig. 1), according to our analyses, probably originated in the Oriental region around 74.9 Ma (Fig. 2a; Table 2), then migrated to the Afrotropical region for the tribes Kallimini, Victorinini and Junoniini, and to Palaearctic and Nearctic region for the Melitaeini. The early lineage of Melitaeini, which apparently colonized the Palaearctic region before 35 Ma (Fig. 2a), diversified and spread from the Old World to the New World during Eocene, which coincided with the early divergence and the dispersal route of key host plants (Asteraceae and Acanthaceae)^23–25^. Our analysis also indicates that *Junonia* and *Melitaea* originated in the Afrotropical region and Palaearctic region, respectively, which was congruent with previous analyses^4, 5^.

Our S-DIVA analysis suggests that the Oriental region was colonized by lineages in Kallimoid clade at least twice from the Afrotropical region: once by the *Hypolimnas* (Junoniini) and once by the *Junonia* (Junoniini), with the two genera subsequently colonizing the Australasian region independently. It is noted that *Hypolimnas* and *Junonia* originated during late Eocene to Oligocene and diversified during Miocene, largely coeval with Miocene Climatic Optimum (18–14 Ma) and the final connection of Arabia with Asia about 21 Ma^26^.

With the dispersal constraints and fossil geographic locations on the related nodes of the dated phylogeny, our analyses suggest several independent entries to the New World from the Old World for Nymphalinae butterflies. These historical biogeographic scenarios can be shown in the reconstruction of the ancestral dispersal routes of the Old Nymphalini, the Victorinini, the Melitaeini, and *Vanessa* with its sister clade, the *Nymphalis*-group (Fig. 2a; Table 2). Based on our time-calibrated phylogeny of Nymphalinae, the time-events curve from our S-DIVA and S-DEC analyses (Fig. 2b) indicates that the frequency of dispersal events may be related with the fluctuations of average global temperature (especially in the Oligocene and Miocene Glaciations) and the dispersals of key host plants (e.g., Acanthaceae and Asteraceae) during middle to late Cenozoic Era, with significantly increased dispersal events during the middle to late Miocene (Fig. 2b), suggesting that the basal clades of Nymphalinae could have undergone several potential extinctions or a relatively long period of ‘stasis’ after the K-Pg mass extinction.

## Discussion

The phylogeny of Nymphalinae remains insufficiently resolved in: 1) the relative position of *Vanessa* and *Hypanartia*, two important lineages for fossil calibration herein, 2) the unstable positions of *Kallimoides*, *Rhinopalpa*, and *Vanessula* and the Kallimini clade, and 3) the robustness of the basal branches in the Nymphalinae trees. We sampled the largest number of species and genera of this subfamily so far to reconstruct the phylogeny by using two nuclear genes, and the Folmer fragment of COI (ca. 650 bp of cytochrome oxidase subunit I, typically standard DNA barcodes) considered of possessing more phylogenetic signal than other mitochondrial gene^27–30^. Our results strongly support the sister relationship between *Vanessa* and the *Nymphalis-*group, and the grouping of the three old-world genera (*Rhinopalpa*, *Kallimoides* and *Vanessula*) within Tribe Victorinini. In addition, our results also support that the Kallimini clade (containing *Catacroptera*, *Kallima* and *Mallika*), one of basal branches in the Nymphalinae tree, was the sister group of Victorinini + Junoniini. The ML and BI analyses with one specimen per species (see Supplementary Table S2) also provided a relatively robust species-level phylogeny underlying the subsequent divergence time estimation and phylogeographic inference.

Divergence time estimation highly depends on tree topology and calibration strategies^31, 32^. In this study, with a wider taxon sampling and relatively highly resolved tree, we used multiple time calibration points, including two fossils and secondary calibrations from the origination of host plants for estimating divergence time of lineages with sparse fossil records in Nymphalinae. Earlier studies show that the two key plant lineages (Acanthaceae and Asteraceae) appeared to have been colonized successively by the clades of subfamily Nymphalinae early in their evolution, rather than after most of their diversification took place^11, 13^. Notably, the glucosinolate-feeding Pierinae diverged from Coliadinae shortly after the appearance of the Brassicales (glucosinolate-containing plants) within 10 million years^12^. Therefore, the appearance times of the host plants are considered valuable proxies for dating relevant lineages in Nymphalinae as maximum time-priors and the host appearance date minus 10 million years as the minimum time-prior. This is a new approach compared with previous studies^2, 3^ using only the host plant maximum constraints.

In order to cross validate the fossil calibration points (*Vanessa amerindica* and *Prodryas persephone*) in our dating procedures, we designed 4 different calibration plans (with or without the time prior for the Kallimoid clade, either the fossil date of *Vanessa amerindica* or that of *Prodryas persephone* used as time prior, see under Methods). We found that Calibration Plans 2 A and 2B yielded the divergence times between *Vanessa* and its sister group (the *Nymphalis*-group) younger than the minimum age of the fossil *Vanessa amerindica* (33.9 Ma) (Table 1), which, we regard, probably indicated that *Prodryas persephone* (related to *Hypanartia*) as a calibration point would potentially cause underestimating the divergence dates in Nymphalini. As for the Calibration Plans 1B and 2B (both without calibration point C4), the posterior divergence times of the Kallimoid clade (node 4, Fig. 1), are ca. 66 Ma and 62 Ma (Table 1), respectively, much younger than the early divergence of the host plant Acanthaceae (ca. 82 Ma^24^). In contrast, with Calibration Plan 1 A (C1, C3 and C4, Fig. 2a), the resulting divergence dates are consistent with the butterfly-hostplant coevolutionary scenarios.

Although the originating area of Nymphalinae (excluding *Historis* and *Baeotus*) before K-Pg event was uncertain, our analyses support previous hypothesis that the Oriental region was the key area for the early divergence of Nymphalinae, especially during the time after the K-Pg mass extinction^3^. The S-DIVA analysis, commonly used for reconstructing wide ancestral ranges on deeper nodes^33, 34^, suggested that the common ancestor of Nymphalini and the Kallimoid clade most likely survived and diverged in the Oriental region (the marginal probability 100%, node 2 in Table 2). It has been shown that some of potential sister groups or paraphyletic groups (Apaturinae, Cyrestinae and Pseudergolinae) were mainly distributed in Oriental region, and similar cases were found in the basal clades of Heliconiinae and Limenitidinae^3, 35^.

The Boreotropics, facilitated intercontinental exchange of tropical biota in the Palaeocene and Eocene (hot and humid climate), and the disruption in the late Eocene-early Oligocene (cooling and drying climate) has proved to play an important role in shaping current tropical disjunct patterns in several vascular plant lineages by vicariance^36–38^. According to our analysis, the Nearctic region was colonized by the ancestor of *Hypanartia* from the Oriental region around 42.9 Ma (Fig. 2a; Table 1), when the extensive frost-free and humid climate belt (the Boreotropics) could facilitate the dispersals across northern mid-latitude areas, and between the northern mid-latitudes and equatorial forests^36^. Later, when the Boreotropics were disrupted in the late Eocene and Oligocene (cooling and drying climate), the ancestor of *Hypanartia* was driven to extinction (the related fossil *Prodryas persephone* found in Colorado) and the distribution area retreated towards the equator while spreading to the South America subsequently. Similar biogeographic pattern was found in Victorinini genera (*Napeocles*, *Siproeta*, *Metamorpha* and *Anartia*) which colonized the Nearctic region by the ‘boreotropical migration’ from the Afrotropical region about 40.5 Ma. Therefore, our results suggest migrational routes for these butterfly groups with independent entries into the New World from the Old World during the middle to late Eocene (see Supplementary Fig. S2).

Based on our new time-calibrated phylogeny, the time-events curve (Fig. 2b) from our S-DIVA and S-DEC analyses shows that the changes in frequency of dispersal events were somewhat correlated with the fluctuations of average global temperature, especially during late Cenozoic (Fig. 2b). It was found that the middle to late Miocene was an important period of accelerated global colonization for lineages of Nymphalinae, though the average global temperature continuously fell after the Miocene Climatic Optimum (18–14 Ma) (Fig. 2b). These global colonization might have been linked to significantly worldwide radiation of the key hostplant Asteraceae during the Neogene^39^, the Old World to New World dispersal events in Acanthaceae within the last 20 million years^24^, and geological events (e.g., the closure of the Tethys Ocean, and the close of the Inter-American Seaway). Similar patterns are found in other flora and fauna^25, 40, 41^.

## Methods

### Molecular dataset, taxon sampling and time-calibration points

We sampled 353 specimens of 269 described species covering 55 of all 56 nymphaline genera (except *Tisona*) (including *Historis*, *Baeotus* and *Pycina* which have contentious position^1^) plus 19 outgroup species (representing all other Nymphalidae subfamilies, see Supplementary Table S1) to reconstruct the specimen-level tree. In addition, based on the topology of specimen-level tree here and previous studies^1, 2, 4, 5, 42^, 144 species (one specimen per species) representing 55 genera of Nymphalinae plus the same 19 outgroup species (see Supplementary Table S2) were selected to reconstruct the Nymphalinae species-level tree, which was subsequently used as blueprint for divergence time estimation. Trees were rooted with *Libythea* for display. For the species-rich and/or worldwidely distributed genera, the basal-most and biogeographically diverse species in the genus were chosen for divergence time estimation and phylogeographic inference according to the specimen-level tree here and previous studies^1, 2, 4, 5, 42^.

The molecular datasets in the present investigation consist of DNA sequences of one mitochondrial gene fragments: cytochrome oxidase subunit I (Folmer fragment of COI, ca. 650 bp), and two nuclear gene fragments: elongation factor 1 alpha (EF-1α, 1038 bp) and wingless (Wg, ca. 380 bp), for a total of 2064 base pairs of concatenated and aligned dataset.

32 new specimens from Palaearctic and Oriental region in China were collected from 2006 to 2012 (Supplementary Table S2). Total genomic DNA was extracted as previously described^43^. All primers, which were used in previous studies^27, 44, 45^, were synthesized by the Sangon Biotechnology Co. Ltd., Shanghai, China.

Each polymerase chain reaction (PCR) was carried out in a final volume of 50 μL, with 0.15 μM of each primer. The PCR settings for COI and wingless gene amplification were adopted as follows: 5 min at 95 °C, followed by 35 cycles of 60 s at 95 °C, 60 s at 47 °C, and 1–2 min at 72 °C. The final elongation step was continued for 10 min at 72 °C. The PCR settings for EF-1α gene amplification were adopted as follows: 5 min at 95 °C, followed by 35 cycles of 60 s at 95 °C, 60 s at 55 °C, and 1–2 min at 72 °C. The final elongation step was also continued for 10 min at 72 °C. The PCR products were purified and sequenced as previously described^43^.

The newly sequenced COI, EF-1α and wingless genes were identified using ClustalX 2.1 software^46^ and the NCBI Internet BLAST search function. The datasets, containing the sequences obtained from earlier studies^1, 2, 11^, were separately aligned according to amino sequence similarity by MUSCLE implied in MEGA5 software^47^ and checked manually. Then the aligned gene sequences were concatenated in DAMBE software^48^. All new sequences were submitted to GenBank (Supplementary Table S2).

The time priors used in the divergence dating here are based on the following fossil dates: *Vanessa amerindica* and *Prodryas persephone* ^49^, both from the Florissant Formation dated at 37.2–33.9 Ma according to Paleobiology Database (www.fossilworks.org), representing the divergence of *Vanessa* and its sister group (Node C1, Fig. 2a) and that between *Hypanartia* and its sister group (Node C2, Fig. 2a), respectively. It is noted that according to the previous study^49^, fossil species *Prodryas persephone* is regarded as a close relative of Genus *Hypanartia*. Both fossils were also used as calibration points in previous studies^2, 3^.

In addition, we used two host-plant dates in constraining the dating procedure as secondary time priors: 49–47.5 Ma based on the new fossil^23^ and estimated divergence time of Asteraceae^50^ as the maximum time prior for the divergence between the *Chlosyne*-group and its sister clade (Node C3, Fig. 2a), and 81.9 Ma based on the crown age of Acanthaceae *s.l*. in a recent study^24^ as the maximun time prior for the Kallimoid clade (Node C4, Fig. 2a).

### Phylogenetic analyses

The substitution saturation of individual gene was test by DAMBE software^48^. Both the Bayesian inference (BI) and maximum likelihood (ML) analyses were used to reconstruct the specimen-level and species-level trees. Besides, maximum parsimony (MP) analyses were used to reconstruct the species-level tree as well.

Bayesian inference (BI) was performed in MrBayes 3.2^51^. For specimen-level tree reconstruction, the PartitionFinder v1.1.1^52^ with user specified scheme (unlinked) was used to choose the best-fit partitioning scheme and models for each subset (see Supplementary Table S3). For the species-level tree reconstruction, the molecular data were treated separately by unpartitioned, mitochondrial and nuclear gene partitioned, and individual gene partitioned, allowing for independent parameter estimates on each partition. The substitution model of each partition was evaluated by jModeltest^53^. In addition, the PartitionFinder v1.1.1^52^ with greedy search scheme (unlinked) was used to choose the best-fit partitioning scheme and models for each subset (see Supplementary Table S3). In the analyses, two simultaneous runs were allowed to go for 2 million generations with sampling each hundredth generation. Each run had four chains, one cold and three heated. When the convergence of MCMC chains was achieved (the average standard deviation of split frequencies (StdDev) <0.01, potential scale reduction factor (PSRF) ≈1), the first 25% of the sampled generations were discarded as burn-in samples. The confidence values of the BI tree were presented as the posterior probabilities (PP).

Maximum likelihood (ML) was performed in raxmlGUI1.5b1^54^, with the same partitioning strategies as performed in BI analysis. The evaluated fit model of GTRGAMMAI to each partition was used and the BS values of the tree were obtained using rapid bootstrapping with 1,000 pseudoreplicates.

Maximum parsimony (MP) was performed in PAUP*4b10^55^ using the tree bisection-reconnection (TBR) branch-swapping algorithm and heuristic search methods. All data were treated as unordered characters and with equal weights. The bootstrap (BS) values were obtained after 500 replicates of heuristic searches and 10 replicates of random stepwise additions of taxa.

### Divergence time analyses

Divergence dates were estimated with the program BEAST v1.8.3^56^, using only the ingroup taxa in the species-level tree (141 species excluding *Historis* + *Baeotus* in Coeini; topology obtained herein) under the uncorrelated lognormal relaxed-clock model. Owing to the higher A-T content and higher evolving rate in the mitochondrial gene, the data were partitioned into the mitochondrial gene region (COI barcodes) and the combined nuclear genes (EF-1a and wingless), with independent GTR + G model. The tree prior was set to the birth–death process with incomplete sampling^57^.

Lognormal priors with soft bounds and confidence intervals were used for calibrations, which allow for incorporation of error around the dating and stratigraphic assignment of the fossils themselves as introduced in previous studies^31, 58, 59^. The time prior with the fossil *Vanessa amerindica* for the divergence between *Vanessa* and the sister group was applied with 95% confidence level for the geological time range of the fossil (37.2–33.9 Ma); same for *Prodryas persephone* as the calibration point for the divergence between *Hypanartia* and its sister group. Both fossils were also used as calibration points in previous studies^2, 3^, but their 95% confidence interval were set here following Tripp & Mcdade^24^.

The two secondary calibration points derived from the recently estimated ages of relevant host plants (maximum 49 Ma for Asteraceae and 81.9 Ma for Acanthaceae *s.l*., see above) were used in this study as the maximum constraint for coevolved butterfly clades, with the minimum time constraint derived by the maximum minus 10 million years as explained under Discussion. The secondary time priors were applied with lognormal distribution with soft bounds at 95% confidence level different from those used in the previous study^3^.

Four different calibration plans were designed herein as follows:

Calibration Plan 1 A: with fossil date of *Vanessa amerindica*, 37.2–33.9 Ma^49^ (C1, Fig. 2a) and two secondary time priors (C3: 49–39 Ma derived from the dates of Asteraceae and C4: 81.9–71.9 Ma derived from the dates of Acanthaceae, Fig. 2a);

Calibration Plan 1B: same as 1 A but without C4;

Calibration Plan 2 A, with fossil date of *Prodryas persephone*, 37.2–33.9 Ma^49^ (C2, Fig. 2a) and two secondary time priors (C3 and C4, Fig. 2a);

Calibration Plan 2B, same as 2 A but without C4.

Additionally, we modified the Calibration Plan 1 A to relax the assumptions of the minimum time priors, by using the same maximum constraints for coevolved butterfly clades, and with the minimum time constraints derived by the maximum minus 5, 15 and 20 m.y., respectively.

In all analyses, we constrained calibrated nodes to be monophyletic as recommended in Drummond *et al*.^58^. Chains were run for 500 million generations, logging every 50,000th generation. To test for convergence, analyses were run until the effective sample sizes of all parameters exceeded 200 and a 10% burn-in was removed. We used Tracer v1.6^60^ to ensure that posterior distributions were sufficiently sampled. Finally, we ran BEAST on 4 calibration plans (1 A, 1B, 2 A and 2B), only from the priors, to explore the influence of the remaining priors on the posterior distribution^58^.

### Ancestral area reconstruction and historical biogeographic inferences

The biogeographic realms generally contained six areas as previously demonstrated^3^: the Neotropical (A), Nearctic (B), Palaearctic (C), Oriental (D), Australasian (E) and Afrotropical (F). We compiled the distribution data for the sampled taxa according to its modern distribution area. For widely distributed genera, multiple species were included and assigned to different regions.

Ancestral areas of internal nodes within trees from the Calibration Plan 1 A were inferred using two strategies implemented in RASP 3.1^61^: (1) enhanced Statistical Dispersal-Vicariance Analysis (S-DIVA), an expansion of event-based Bayes-DIVA which explicitly utilizes an entire posterior distribution of trees to account for both phylogenetic uncertainty and uncertainty in ancestral states^61^; and (2) Lagrange Analyses using Dispersal-Extinction-Cladogenesis (DEC) model and Statistical DEC (Bays-Lagrange, S-DEC) model, which accommodate differing dispersal probabilities among areas across different time-slices and can integrate branch lengths, divergence times, and geological information. For all analyses the maximum number of ancestral areas was constrained to two, reflecting the assumption that the ranges of ancestral nymphalid species were similar to those of their extant descendants.

In S-DIVA analysis, random 1,000 BEAST output trees and the maximum clade credibility tree were used. The number of reconstructions kept for each tree in tree dataset was set to 100 and maximum reconstructions for final tree was set to 1,000. In Lagrange Analyses, the matrix parameters of dispersal rates were set according to, on one hand, the connection between areas in different times, e.g., the connection of the African and Asian continents about 21 Ma, the presence of a peninsula (GAARlandia) about 35–33 Ma, and the connection of Africa with Europe (subsequently Oriental region) about 60 Ma^26, 62–65^; and on the other hand, the biological interchange between South America and Africa until Eocene^23^. The constraints on rates of dispersal between areas were implemented in five time slices, and the parameters were based on previous study^66^ (see Supplementary Table S5); some ancestral distributions of clades were specified based on the fossils of related species reported in previous studies^1, 3^; the time-events curve was calculated by the Time option in RASP^61^.

## Electronic supplementary material

Supplementary Information
