# Supplementary material for: Dated phylogeny and dispersal history of the butterfly subfamily Nymphalinae (Lepidoptera: Nymphalidae)
Source: Sci Rep. 2017 Aug 18;7:8799. doi: 10.1038/s41598-017-08993-w (PMC5562872; doi:10.1038/s41598-017-08993-w)

**Supplementary Material**

**Dated phylogeny and dispersal history of the butterfly subfamily Nymphalinae (Lepidoptera: Nymphalidae)**

Chengyong Su, Qinghui Shi, Xiaoyan Sun, Junye Ma, Chunxiang Li, Jiasheng Hao and Qun Yang

**Contents**

**Supplementary Table S1.** Taxon sampling in specimen-level tree with GenBank accession number.

**Supplementary Table S2.** Taxon sampling in species-level tree with GenBank accession number and main distribution.

**Supplementary Table S3.** Substitution model of each partition with different partitioning schemes.

**Supplementary Table S4.** Comparison of crown ages of major lineages and the most recent common ancestor (MRCA) of major splits in Nymphalinae under the modified Calibration Plan 1A.

**Supplementary Table S5.** Dispersal rate scaling matrices used in Lagrange analyses.

**Supplementary Figure S1.** The species-level phylogenetic relationships of Nymphalinae based on maximum likelihood and Bayesian analyses.

**Supplementary Figure S2.** Inter-continental routes of dispersal for major clades of Nymphalinae.

**Supplementary Table S1.** Taxon sampling (*new sampled specimen) in specimen-level tree with GenBank accession number. For different specimens of one species, the number in the Voucher Code followed the name of species.

| **number** | **Species** | **Genbank accession number** | | |
| --- | --- | --- | --- | --- |
|  |  | ***COI*** | ***EF1-α*** | ***wingless*** |
| 1 | *Aglais io 63-16* | AY248785 | AY248810 | AF412766 |
| 2 | *Aglais io** | KX824672 | KX824704 | KX824736 |
| 3 | *Aglais milberti* | AY248787 | AY248812 | AY248828 |
| 4 | *Aglais urticae 63-3* | AY248786 | AY248811 | AF412777 |
| 5 | *Aglais urticae** | KX824675 | KX824707 | KX824739 |
| 6 | *Anartia amathea* | AY788606 | AY788708 | AY788469 |
| 7 | *Anartia fatima* | AY788607 | AY788709 | AY788470 |
| 8 | *Anartia jatrophae* | AY788608 | AY788710 | AY788471 |
| 9 | *Antanartia delius* | AY788610 | AY788712 | AY788473 |
| 10 | *Antanartia schaenia* | AY218236 | AY218255 | AF412780 |
| 11 | *Anthanassa ardys* | AF187743 | AY788713 | AY788474 |
| 12 | *Anthanassa drusilla alecta* | AY788611 | AY788714 | AY788475 |
| 13 | *Anthanassa frisia hermas* | EF493929 | EF493977 | EF493870 |
| 14 | *Anthanassa frisia tulcis 104-12* | AY788612 | AY788717 | AY788478 |
| 15 | *Anthanassa frisia tulcis 34-8* | AF187802 | EF493978 | EF493871 |
| 16 | *Anthanassa otanes* | AF187790 | AY788715 | AY788476 |
| 17 | *Anthanassa texana* | AF187806 | AY788716 | AY788477 |
| 18 | *Antillea pelops* | GQ864733 | GQ864827 | GQ864421 |
| 19 | *Antillea proclea* | EF493928 | EF493976 | EF493869 |
| 20 | *Araschnia burejana** | KX824689 | KX824721 | KX824753 |
| 21 | *Araschnia dohertyi ** | KX824690 | KX824722 | KX824754 |
| 22 | *Araschnia doris** | KX824691 | KX824723 | KX824755 |
| 23 | *Araschnia levana* | AY248780 | AY248805 | AF412762 |
| 24 | *Araschnia prorsoides** | KX824692 | KX824724 | KX824756 |
| 25 | *Atlantea pantoni* | GQ864741 | GQ864835 | GQ864429 |
| 26 | *Baeotus aelius* | AY788613 | AY788718 | AY788479 |
| 27 | *Baeotus amazonicus* | AY788614 | AY788719 | AY788480 |
| 28 | *Baeotus beotus* | AY788615 | AY788720 | AY788481 |
| 29 | *Baeotus deucalion* | AY788616 | AY788721 | AY788482 |
| 30 | *Castilia castilla occidentalis* | EF493930 | EF493979 | EF493872 |
| 31 | *Castilia eranites* | AY788617 | AY788722 | AY788483 |
| 32 | *Castilia myia* | AF187784 | EF493980 | EF493873 |
| 33 | *Castilia ofella* | AY788618 | AY788723 | AY788484 |
| 34 | *Castilia perilla* | EF493931 | EF493981 | EF493874 |
| 35 | *Catacroptera cloanthe* | AY788619 | AY788724 | AY788485 |
| 36 | *Chlosyne acastus* | AF187735 | AY788725 | AY788486 |
| 37 | *Chlosyne cyneas* | AF187757 | AY788726 | AY788487 |
| 38 | *Chlosyne gaudialis* | AF187770 | AY788727 | AY788488 |
| 39 | *Chlosyne gorgone* | AF187772 | AY788728 | AY788489 |
| 40 | *Chlosyne harrisii* | AF187773 | AY788729 | AY788490 |
| 41 | *Chlosyne janais* | AY788620 | AY788730 | AY788491 |
| 42 | *Chlosyne lacinia* | AY090227 | AY090195 | AY090161 |
| 43 | *Chlosyne narva* | AF187786 | AY788731 | AY788492 |
| 44 | *Chlosyne nycteis* | AF187788 | AY788732 | AY788493 |
| 45 | *Chlosyne palla* | AF187791 | AY788733 | AY788494 |
| 46 | *Chlosyne theona* | AF187808 | AY788734 | AY788495 |
| 47 | *Colobura dirce* | AY090228 | AY090196 | AY090162 |
| 48 | *Dagon pusillus* | EF493932 | EF493982 | EF493875 |
| 49 | *Doleschallia bisaltide 64-5* | AY788621 | AY788735 | AY788496 |
| 50 | *Doleschallia bisaltide** | KX824663 | KX824695 | KX824727 |
| 51 | *Dymasia dymas* | AF187764 | AY788785 | AY788545 |
| 52 | *Eresia carme* | EF493935 | EF493985 | EF493878 |
| 53 | *Eresia casiphia* | EF493936 | EF493986 | EF493879 |
| 54 | *Eresia clio* | AY788622 | AY788736 | AY788497 |
| 55 | *Eresia coela* | AY788623 | AY788737 | AY788498 |
| 56 | *Eresia datis phaedima* | EF493942 | EF493992 | EF493885 |
| 57 | *Eresia eunice* | AY788624 | AY788738 | AY788499 |
| 58 | *Eresia ithomioides alsina* | EF493933 | EF493983 | EF493876 |
| 59 | *Eresia ithomioides eutropia 104-7* | EF493940 | EF493990 | EF493883 |
| 60 | *Eresia ithomioides eutropia 120-17* | EF493937 | EF493987 | EF493880 |
| 61 | *Eresia lansdorfi* | EF493938 | EF493988 | EF493881 |
| 62 | *Eresia letitia* | AY788625 | AY788739 | AY788500 |
| 63 | *Eresia levina* | EF493939 | EF493989 | EF493882 |
| 64 | *Eresia nauplius plagiata* | EF493944 | EF493994 | EF493887 |
| 65 | *Eresia pelonia* | AY788626 | AY788740 | AY788501 |
| 66 | *Eresia perna perna* | EF493941 | EF493991 | EF493884 |
| 67 | *Eresia phillyra phillyra* | EF493943 | EF493993 | EF493886 |
| 68 | *Eresia polina polina* | EF493945 | EF493995 | EF493888 |
| 69 | *Eresia quintilla* | AY788627 | AY788741 | AY788502 |
| 70 | *Eresia sestia* | AY788628 | AY788742 | AY788503 |
| 71 | *Eresia sticta* | EF493946 | EF493996 | EF493889 |
| 72 | *Euphydryas aurinia* | AF187746 | AY788743 | AY788504 |
| 73 | *Euphydryas chalcedona* | AF187752 | AY788744 | AY788505 |
| 74 | *Euphydryas desfontainii* | AY090226 | AY090193 | AY090159 |
| 75 | *Euphydryas editha* | AF187765 | AY788745 | AY788506 |
| 76 | *Euphydryas gillettii* | AF187771 | AY788746 | AY788507 |
| 77 | *Euphydryas intermedia** | KX824671 | KX824703 | KX824735 |
| 78 | *Euphydryas phaeton* | AF187797 | AY788747 | AY788508 |
| 79 | *Gnathotriche exclamationis* | AY788629 | AY788748 | AY788509 |
| 80 | *Gnathotriche mundina* | EF493927 | EF493975 | EF493868 |
| 81 | *Higginsius fasciatus* | AY788630 | AY788749 | AY788510 |
| 82 | *Historis acheronta* | AY788631 | AY788750 | AY788511 |
| 83 | *Historis odius* | AY788632 | AY788751 | AY788512 |
| 84 | *Hypanartia bella* | AY788638 | AY788757 | AF246590 |
| 85 | *Hypanartia charon* | AY788639 | AY788758 | AY788518 |
| 86 | *Hypanartia kefersteinii* | AY788640 | AY788759 | AY788519 |
| 87 | *Hypanartia lethe* | AF187774 | AY788760 | AY788520 |
| 88 | *Hypanartia lindigii* | AY248781 | AY248806 | AF412759 |
| 89 | *Hypolimnas alimena* | AY788633 | AY788752 | AY788513 |
| 90 | *Hypolimnas anthedon* | AY788634 | AY788753 | AY788514 |
| 91 | *Hypolimnas bolina 29-5* | AF187775 | AY248802 | AF412775 |
| 92 | *Hypolimnas bolina** | KX824661 | KX824693 | KX824725 |
| 93 | *Hypolimnas bolina62-6* | AY090224 | AY090190 | AY090156 |
| 94 | *Hypolimnas misippus 68-3* | AY788635 | AY788754 | AY788515 |
| 95 | *Hypolimnas missipus** | KX824662 | KX824694 | KX824726 |
| 96 | *Hypolimnas pandarus* | AY788636 | AY788755 | AY788516 |
| 97 | *Hypolimnas usambara* | AY788637 | AY788756 | AY788517 |
| 98 | *Janatella fellula* | EF493947 | EF493997 | EF493890 |
| 99 | *Janatella hera* | EF493973 | EF494032 | EF493925 |
| 100 | *Janatella leucodesma* | AY788641 | AY788761 | AY788521 |
| 101 | *Junonia almana 131-7* | EU053287 | EU053323 | EU053360 |
| 102 | *Junonia almana 131-8* | EU053288 | EU053324 | EU053361 |
| 103 | *Junonia almana 29-3* | EU053289 | EU053325 | EU053362 |
| 104 | *Junonia almana** | KX824683 | KX824715 | KX824747 |
| 105 | *Junonia ansorgei* | EU053290 | EU053326 | EU053363 |
| 106 | *Junonia artaxia* | AY788642 | AY788762 | AY788522 |
| 107 | *Junonia atlites 131-9* | EU053291 | EU053327 | EU053364 |
| 108 | *Junonia atlites 29-6* | EU053292 | EU053328 | EU053365 |
| 109 | *Junonia atlites 78-4* | EU053293 | EU053329 | EU053366 |
| 110 | *Junonia atlites 98-2* | EU053294 | EU053330 | EU053367 |
| 111 | *Junonia atlites** | KX824684 | KX824716 | KX824748 |
| 112 | *Junonia coenia 38-18* | AY248777 | EU053331 | EU053368 |
| 113 | *Junonia coenia 85-13* | AY788643 | AY248801 | AY248826 |
| 114 | *Junonia cymodoce* | AY788652 | AY788771 | AY788531 |
| 115 | *Junonia erigone* | AY788644 | AY788763 | AY788523 |
| 116 | *Junonia evarete 126-20* | EU053295 | EU053332 | EU053369 |
| 117 | *Junonia evarete 129-30* | EU053296 | EU053333 | EU053370 |
| 118 | *Junonia evarete 136-17* | EU053297 | EU053334 | EU053371 |
| 119 | *Junonia evarete 36-2* | EU053298 | EU053335 | EU053372 |
| 120 | *Junonia evarete 84-15* | EU053299 | EU053336 | EU053373 |
| 121 | *Junonia genoveva* | EU053300 | EU053337 | EU053374 |
| 122 | *Junonia gregorii* | EU053301 | EU053338 | EU053375 |
| 123 | *Junonia hedonia 33-2* | EU053302 | EU053339 | EU053376 |
| 124 | *Junonia hedonia 78-5* | EU053303 | EU053340 | EU053377 |
| 125 | *Junonia hierta 101-16* | EU053304 | EU053341 | EU053378 |
| 126 | *Junonia hierta 80-6* | EU053305 | EU053342 | EU053379 |
| 127 | *Junonia hierta 88-10* | EU053306 | EU053343 | EU053380 |
| 128 | *Junonia hierta 88-12* | EU053307 | EU053344 | EU053381 |
| 129 | *Junonia hierta** | KX824685 | KX824717 | KX824749 |
| 130 | *Junonia iphita 68-17* | AY090225 | AY090191 | AY090157 |
| 131 | *Junonia iphita** | KX824686 | KX824718 | KX824750 |
| 132 | *Junonia lemonias 81-4* | EU053309 | EU053346 | EU053383 |
| 133 | *Junonia lemonias 97-10* | EU053311 | EU053348 | EU053385 |
| 134 | *Junonia lemonias 97-9* | EU053310 | EU053347 | EU053384 |
| 135 | *Junonia lemonias** | KX824687 | KX824719 | KX824751 |
| 136 | *Junonia lemonias101-15* | EU053308 | EU053345 | EU053382 |
| 137 | *Junonia natalica 68-13* | AY788645 | AY788764 | AY788524 |
| 138 | *Junonia natalica 83-11* | EU053312 | EU053349 | EU053386 |
| 139 | *Junonia natalica 88-13* | EU053313 | EU053350 | EU053387 |
| 140 | *Junonia oenone* | AY788646 | AY788765 | AY788525 |
| 141 | *Junonia orithya* | EU053314 | EU053351 | EU053388 |
| 142 | *Junonia sophia* | AY788647 | AY788766 | AY788526 |
| 143 | *Junonia terea 68-15* | AY788648 | AY788767 | AY788527 |
| 144 | *Junonia terea 82-14* | EU053317 | EU053354 | EU053391 |
| 145 | *Junonia terea 83-3* | EU053318 | EU053355 | EU053392 |
| 146 | *Junonia touhilimasa* | AY788649 | AY788768 | AY788528 |
| 147 | *Junonia villida 99-1* | EU053321 | EU053358 | EU053395 |
| 148 | *Junonia villida 99-2* | EU053322 | EU053359 | EU053396 |
| 149 | *Junonia westermanni 82-11* | EU053319 | EU053356 | EU053393 |
| 150 | *Junonia westermanni 83-4* | EU053320 | EU053357 | EU053394 |
| 151 | *Kallima inachus 85-15* | AY788650 | AY788769 | AY788529 |
| 152 | *Kallima inachus** | KX824673 | KX824705 | KX824737 |
| 153 | *Kallima paralekta* | AY090229 | AY090197 | AY090163 |
| 154 | *Kallimoides rumia* | AY788651 | AY788770 | AY788530 |
| 155 | *Kaniska canace 164-1* | FJ639397 | FJ639493 | FJ639395 |
| 156 | *Kaniska canace 19-11* | AY248792 | AY248817 | AY248833 |
| 157 | *Kaniska canace** | KX824674 | KX824706 | KX824738 |
| 158 | *Mallika jacksoni* | AY788653 | AY788772 | AY788532 |
| 159 | *Mazia amazonica* | AY788654 | AY788773 | AY788533 |
| 160 | *Melitaea acraeina* | FJ462229 | FJ462289 | FJ462164 |
| 161 | *Melitaea aetherie* | FJ462230 | FJ462290 | FJ462165 |
| 162 | *Melitaea ala* | FJ462231 | FJ462291 | FJ462166 |
| 163 | *Melitaea ambigua* | AF187736 | FJ462292 | FJ462167 |
| 164 | *Melitaea ambrisia* | FJ462232 | FJ462293 | FJ462168 |
| 165 | *Melitaea amoenula* | AF187737 | FJ462294 | FJ462169 |
| 166 | *Melitaea arcesia* | AF187741 | FJ462295 | FJ462170 |
| 167 | *Melitaea arduinna* | AF187742 | AY788774 | AY788534 |
| 168 | *Melitaea athalia* | FJ462234 | FJ462297 | FJ462171 |
| 169 | *Melitaea athene* | AF187799 | FJ462298 | FJ462172 |
| 170 | *Melitaea aurelia* | AF187745 | FJ462299 | FJ462173 |
| 171 | *Melitaea bellona* | FJ462236 | FJ462301 | FJ462174 |
| 172 | *Melitaea britomartis 15-13* | AF187748 | FJ462302 | FJ462175 |
| 173 | *Melitaea britomartis 69-8* | AY788655 | AY788775 | AY788535 |
| 174 | *Melitaea cassandra* | FJ462237 | FJ462303 | FJ462176 |
| 175 | *Melitaea casta* | FJ462238 | FJ462304 | FJ462177 |
| 176 | *Melitaea caucasogenita* | FJ462239 | FJ462305 | FJ462178 |
| 177 | *Melitaea celadussa* | FJ462240 | FJ462306 | FJ462179 |
| 178 | *Melitaea centralasiae* | FJ462241 | FJ462307 | FJ462180 |
| 179 | *Melitaea chitralensis* | FJ462242 | FJ462308 | FJ462181 |
| 180 | *Melitaea chuana* | FJ462243 | FJ462309 | FJ462182 |
| 181 | *Melitaea cinxia* | AY788656 | AY788776 | AY788536 |
| 182 | *Melitaea collina* | FJ462244 | FJ462311 | FJ462183 |
| 183 | *Melitaea consulis* | FJ462245 | FJ462312 | FJ462184 |
| 184 | *Melitaea deione 126* | FJ462246 | FJ462313 | FJ462186 |
| 185 | *Melitaea deione 150-13* | FJ462247 | FJ462314 | FJ462185 |
| 186 | *Melitaea deione 95-5* | AY788657 | AY788777 | AY788537 |
| 187 | *Melitaea deserticola 3-10* | FJ462248 | FJ462316 | FJ462188 |
| 188 | *Melitaea deserticola 34-12* | AF187759 | FJ462315 | FJ462187 |
| 189 | *Melitaea diamina 10-24* | AF187761 | FJ462317 | FJ462189 |
| 190 | *Melitaea diamina** | KX824680 | KX824712 | KX824744 |
| 191 | *Melitaea didyma 107-5* | FJ462253 | FJ462322 | FJ462194 |
| 192 | *Melitaea didyma 3-3* | FJ462250 | FJ462319 | FJ462191 |
| 193 | *Melitaea didyma 6-7* | FJ462251 | FJ462320 | FJ462192 |
| 194 | *Melitaea didyma 7-8* | FJ462252 | FJ462321 | FJ462193 |
| 195 | *Melitaea didyma 99-12* | FJ462249 | FJ462318 | FJ462190 |
| 196 | *Melitaea didymoides 26-1* | AF187762 | AY090194 | AY090160 |
| 197 | *Melitaea didymoides 28-14* | FJ462254 | FJ462323 | FJ462195 |
| 198 | *Melitaea didymoides** | KX824681 | KX824713 | KX824745 |
| 199 | *Melitaea elizabethae* | FJ462255 | FJ462324 | FJ462196 |
| 200 | *Melitaea enarea* | FJ462256 | FJ462325 | FJ462197 |
| 201 | *Melitaea fergana* | FJ462257 | FJ462326 | FJ462198 |
| 202 | *Melitaea interrupta* | FJ462260 | FJ462328 | FJ462200 |
| 203 | *Melitaea latonigena 25-3* | AF187780 | AY788778 | AY788538 |
| 204 | *Melitaea latonigena* | FJ462261 | FJ462329 | FJ462201 |
| 205 | *Melitaea leechi* | FJ462262 | FJ462330 | FJ462202 |
| 206 | *Melitaea ludmilla* | FJ462263 | FJ462331 | FJ462203 |
| 207 | *Melitaea lunalata* | FJ462265 | FJ462333 | FJ462205 |
| 208 | *Melitaea maracandica* | FJ462266 | FJ462334 | FJ462206 |
| 209 | *Melitaea menetriesi* | FJ462267 | FJ462335 | FJ462207 |
| 210 | *Melitaea minerva* | FJ462268 | FJ462336 | FJ462208 |
| 211 | *Melitaea ninae* | FJ462269 | FJ462337 | FJ462209 |
| 212 | *Melitaea pallas* | FJ462270 | FJ462338 | FJ462210 |
| 213 | *Melitaea parthenoides* | FJ462271 | FJ462339 | FJ462211 |
| 214 | *Melitaea permuta* | FJ462272 | FJ462340 | FJ462212 |
| 215 | *Melitaea persea 120-11* | FJ462273 | FJ462341 | FJ462213 |
| 216 | *Melitaea persea 34-10* | AF187796 | AY788779 | AY788539 |
| 217 | *Melitaea phoebe 15-14* | FJ462274 | FJ462342 | FJ462214 |
| 218 | *Melitaea phoebe 6-6* | FJ462275 | FJ462343 | FJ462215 |
| 219 | *Melitaea plotina* | FJ462277 | FJ462345 | FJ462217 |
| 220 | *Melitaea protomedia 40-6* | FJ462278 | FJ462346 | FJ462218 |
| 221 | *Melitaea protomedia** | KX824682 | KX824714 | KX824746 |
| 222 | *Melitaea punica 34-11* | AF187803 | AY788781 | AY788541 |
| 223 | *Melitaea punica 3-7* | FJ462276 | FJ462344 | FJ462216 |
| 224 | *Melitaea romanovi* | FJ462280 | FJ462348 | FJ462220 |
| 225 | *Melitaea saxatilis* | FJ462281 | FJ462349 | FJ462221 |
| 226 | *Melitaea scotosia* | AF187804 | AY788780 | AY788540 |
| 227 | *Melitaea shandura* | FJ462282 | FJ462350 | FJ462222 |
| 228 | *Melitaea sibina* | FJ462283 | FJ462351 | FJ462223 |
| 229 | *Melitaea solona* | FJ462284 | FJ462352 | FJ462224 |
| 230 | *Melitaea sultanensis* | FJ462285 | FJ462353 | FJ462225 |
| 231 | *Melitaea sutschana* | AF187805 | FJ462354 | FJ462226 |
| 232 | *Melitaea telona* | FJ462279 | FJ462347 | FJ462219 |
| 233 | *Melitaea trivia* | AF187810 | AY788782 | AY788542 |
| 234 | *Melitaea varia* | AF187812 | AY788783 | AY788543 |
| 235 | *Melitaea wiltshirei* | FJ462288 | FJ462356 | FJ462228 |
| 236 | *Metamorpha elissa* | AY788658 | AY788784 | AY788544 |
| 237 | *Microtia elva* | AY788660 | AY788787 | AY788547 |
| 238 | *Mynes geoffroyi* | AY248778 | AY248803 | AF412760 |
| 239 | *Napeocles jucunda* | AY788661 | AY788788 | AY788548 |
| 240 | *Nymphalis antiopa 70-2* | AY218246 | AY218266 | AY218284 |
| 241 | *Nymphalis antiopa** | KX824668 | KX824700 | KX824732 |
| 242 | *Nymphalis californica* | AY248789 | AY248814 | AY248830 |
| 243 | *Nymphalis l-album 78-1* | AY248791 | AY248816 | AY248832 |
| 244 | *Nymphalis polychloros* | AY248788 | AY248813 | AY248829 |
| 245 | *Nymphalis vau-album** | KX824669 | KX824701 | KX824733 |
| 246 | *Nymphalis xanthomelas 84-1* | AY248790 | AY248815 | AY248831 |
| 247 | *Nymphalis xanthomelas** | KX824670 | KX824702 | KX824734 |
| 248 | *Ortilia dicoma* | EF493948 | EF493998 | EF493891 |
| 249 | *Ortilia gentina* | EF493950 | EF494000 | EF493893 |
| 250 | *Ortilia ithra* | EF493949 | EF493999 | EF493892 |
| 251 | *Ortilia liriope* | EF493972 | EF494031 | EF493924 |
| 252 | *Ortilia orthia* | EF493951 | EF494001 | EF493894 |
| 253 | *Ortilia orticas orticas* | EF493952 | EF494002 | EF493895 |
| 254 | *Ortilia velica 106-5* | EF493954 | EF494004 | EF493897 |
| 255 | *Ortilia velica 114-7* | EF493953 | EF494003 | EF493896 |
| 256 | *Phyciodes batesii apsaalooke* | AY156596 | EF494006 | EF493899 |
| 257 | *Phyciodes batesii lakota* | AF187747 | EF494005 | EF493898 |
| 258 | *Phyciodes batesii maconensis* | AY156601 | EF494007 | EF493900 |
| 259 | *Phyciodes cocyta* | AF187755 | AY090192 | AY090158 |
| 260 | *Phyciodes cocyta selenis 11-5* | AY156606 | EF494008 | EF493901 |
| 261 | *Phyciodes cocyta selenis 47-12* | AY156608 | EF494009 | EF493902 |
| 262 | *Phyciodes graphica vesta* | AY156684 | AY788790 | AY788550 |
| 263 | *Phyciodes mylitta* | AF187785 | AY788791 | AY788551 |
| 264 | *Phyciodes orseis* | AY156631 | AY788792 | AY788552 |
| 265 | *Phyciodes pallescens* | AY156640 | AY788793 | AY788553 |
| 266 | *Phyciodes pallida 34-6* | AF187792 | AY788794 | AY788554 |
| 267 | *Phyciodes pallida 58-5* | AY156637 | EF494011 | EF493904 |
| 268 | *Phyciodes phaon* | AF187798 | AY788795 | AY788555 |
| 269 | *Phyciodes phaon phaon* | AY156638 | EF494012 | EF493905 |
| 270 | *Phyciodes picta* | AF187800 | AY788796 | AY788556 |
| 271 | *Phyciodes pulchella camillus* | AY156643 | EF494013 | EF493906 |
| 272 | *Phyciodes pulchella pulchella* | AY156662 | AY788797 | AY788557 |
| 273 | *Phyciodes tharos* | AF187807 | AY788798 | AY788558 |
| 274 | *Phyciodes tharos tharos* | EF493955 | EF494010 | EF493903 |
| 275 | *Phystis simois simois* | EF493956 | EF494014 | EF493907 |
| 276 | *Poladryas arachne* | AF187740 | AY788799 | AY788559 |
| 277 | *Polygonia c-album 70-3* | AY090222 | AY090188 | AY090154 |
| 278 | *Polygonia c-album** | KX824664 | KX824696 | KX824728 |
| 279 | *Polygonia c-aureum 65-8* | AY248799 | AY248824 | AF412786 |
| 280 | *Polygonia c-aureun** | KX824665 | KX824697 | KX824729 |
| 281 | *Polygonia comma* | AY248794 | AY248819 | AF412781 |
| 282 | *Polygonia egea* | AY248800 | AY248825 | AY248838 |
| 283 | *Polygonia faunus* | AY248798 | AY248823 | AY248837 |
| 284 | *Polygonia gracilis* | AY248797 | AY248822 | AY248836 |
| 285 | *Polygonia haroldi* | AY788662 | AY788800 | AY788560 |
| 286 | *Polygonia interrogationis* | AY248793 | AY248818 | AY248834 |
| 287 | *Polygonia oreas* | AY788663 | AY788801 | AY788561 |
| 288 | *Polygonia progne* | AY248795 | AY248820 | AF412765 |
| 289 | *Polygonia satyrus* | AY248796 | AY248821 | AY248835 |
| 290 | *Precis andremiaja* | AY788664 | AY788802 | AY788562 |
| 291 | *Precis antilope* | AY788665 | AY788803 | AY788563 |
| 292 | *Precis archesia* | AY788666 | AY788804 | AY788564 |
| 293 | *Precis ceryne* | AY788667 | AY788805 | AY788565 |
| 294 | *Precis cuama* | AY788668 | AY788806 | AY788566 |
| 295 | *Precis octavia* | AY788669 | AY788807 | AY788567 |
| 296 | *Precis sinuata* | AY788670 | AY788808 | AY788568 |
| 297 | *Precis tugela* | AY788671 | AY788809 | AY788569 |
| 298 | *Protogoniomorpha anacardii* | AY090223 | AY090189 | AY090155 |
| 299 | *Protogoniomorpha cytora* | AY788672 | AY788810 | AY788570 |
| 300 | *Protogoniomorpha parhassus* | AY788673 | AY788811 | AY788571 |
| 301 | *Pycina zamba* | GQ864804 | GQ864898 | GQ864492 |
| 302 | *Rhinopalpa polynice* | AY788674 | AY788812 | AY788572 |
| 303 | *Salamis anteva* | AY788675 | AY788813 | AY788573 |
| 304 | *Salamis cacta* | AY788676 | AY788814 | AY788574 |
| 305 | *Siproeta epaphus* | AY788677 | AY788815 | AY788575 |
| 306 | *Siproeta stelenes* | AY218248 | AY218268 | AY218286 |
| 307 | *Smyrna blomfildia* | AY788678 | AY788816 | AY788576 |
| 308 | *Symbrenthia brabira** | KX824676 | KX824708 | KX824740 |
| 309 | *Symbrenthia hypatia* | AY248779 | AY248804 | AF412784 |
| 310 | *Symbrenthia hypselis 97-2* | AY788680 | AY788818 | AY788578 |
| 311 | *Symbrenthia hypselis** | KX824677 | KX824709 | KX824741 |
| 312 | *Symbrenthia lilea 97-3* | AY788679 | AY788817 | AY788577 |
| 313 | *Symbrenthia lilea** | KX824678 | KX824710 | KX824742 |
| 314 | *Symbrenthia niphanda** | KX824679 | KX824711 | KX824743 |
| 315 | *Tegosa anieta* | AY788681 | AY788819 | AY788579 |
| 316 | *Tegosa claudina 108-9* | EF493957 | EF494015 | EF493908 |
| 317 | *Tegosa claudina 116-21* | EF493958 | EF494016 | EF493909 |
| 318 | *Tegosa claudina 124-12* | EF493960 | EF494018 | EF493911 |
| 319 | *Tegosa claudina 124-19* | EF493959 | EF494017 | EF493910 |
| 320 | *Tegosa etia* | EF493961 | EF494019 | EF493912 |
| 321 | *Tegosa infrequens 122-23* | EF493963 | EF494021 | EF493914 |
| 322 | *Tegosa infrequens 92-7* | EF493962 | EF494020 | EF493913 |
| 323 | *Tegosa orobia orobia* | EF493964 | EF494022 | EF493915 |
| 324 | *Tegosa orobia ursula* | EF493967 | EF494025 | EF493918 |
| 325 | *Tegosa selene* | EF493965 | EF494023 | EF493916 |
| 326 | *Tegosa similis* | EF493966 | EF494024 | EF493917 |
| 327 | *Tegosa tissoides* | AY788682 | AY788820 | AY788580 |
| 328 | *Telenassa berenice drusinilla* | EF493968 | EF494026 | EF493919 |
| 329 | *Telenassa delphia nana* | EF493970 | EF494029 | EF493922 |
| 330 | *Telenassa fontus* | EF493974 | EF494033 | EF493926 |
| 331 | *Telenassa teletusa burchelli 115-7* | EF493969 | EF494027 | EF493920 |
| 332 | *Telenassa teletusa burchelli 38-5* | AF187749 | EF494028 | EF493921 |
| 333 | *Telenassa teletusa teletusa* | EF493971 | EF494030 | EF493923 |
| 334 | *Telenassa trimaculata* | AY788683 | AY788821 | AY788581 |
| 335 | *Texola elada* | AY788659 | AY788786 | AY788546 |
| 336 | *Tigridia acesta* | AY788684 | AY788822 | AY788582 |
| 337 | *Vanessa abyssinica* | AY788609 | AY788711 | AY788472 |
| 338 | *Vanessa annabella* | AY788685 | AY788823 | AY788583 |
| 339 | *Vanessa atalanta* | AY090221 | AY090187 | AF412772 |
| 340 | *Vanessa braziliensis* | AY788686 | AY788824 | AY788584 |
| 341 | *Vanessa cardui 63-3* | AY248782 | AY248807 | AF412770 |
| 342 | *Vanessa cardui** | KX824666 | KX824698 | KX824730 |
| 343 | *Vanessa gonerilla* | AY248784 | AY248809 | AF412782 |
| 344 | *Vanessa indica 63-9* | AY788687 | AY788825 | AY788585 |
| 345 | *Vanessa indica** | KX824667 | KX824699 | KX824731 |
| 346 | *Vanessa itea* | AY788688 | AY788826 | AY788586 |
| 347 | *Vanessa kershawi* | AY788689 | AY788827 | AY788587 |
| 348 | *Vanessa myrinna* | AY788690 | AY788828 | AY788588 |
| 349 | *Vanessa virginiensis* | AY248783 | AY248808 | AY248827 |
| 350 | *Vanessula milca* | AY788691 | AY788829 | AY788589 |
| 351 | *Yoma algina* | AY788692 | AY788830 | AY788590 |
| 352 | *Yoma sabina 132-2* | EU053399 | EU053397 | EU053398 |
| 353 | *Yoma sabina** | KX824688 | KX824720 | KX824752 |
|  | **Outgroup** |  |  |  |
| 354 | *Cyrestis thyodamas* | AY218240 | AY218260 | AY218278 |
| 355 | *Heliconius hecale* | AY090202 | AY090168 | AY090135 |
| 356 | *Adelpha bredowii* | AY788591 | AY788693 | AY788457 |
| 357 | *Amnosia decora* | AY218235 | AY218254 | AY218273 |
| 358 | *Dichorragia nesimachus* | AY788602 | AY788704 | AY788466 |
| 359 | *Pseudergolis wedah* | AY788605 | AY788707 | AY788468 |
| 360 | *Ariadne enotrea* | AY218237 | AY218256 | AY218274 |
| 361 | *Byblia anvatara* | AY788595 | AY788697 | AY788460 |
| 362 | *Asterocampa leilia* | AF187734 | AY218257 | AY218275 |
| 363 | *Eulaceura osteria* | AY788593 | AY788695 | AF246588 |
| 364 | *Mimathyma schrenckii* | AY788594 | AY788696 | AY788459 |
| 365 | *Libytheana carinenta* | GQ864786 | GQ864880 | GQ864474 |
| 366 | *Libytheana terena* | EU141364 | EU136671 | EU141245 |
| 367 | *Libythea myrrha* | KT286524 | KT286217 | KT286044 |
| 368 | *Danaus chrysippus* | KP007638 | JX185963 | KP007893 |
| 369 | *Danaus plexippus* | DQ018954 | DQ018921 | AF246564 |
| 370 | *Euploea mulciber* | KM102678 | GU365940 | GU365959 |
| 371 | *Eumenis autonoe* | GU372518 | GU372609 | KM200294 |
| 372 | *Melanitis leda* | AY090207 | AY090173 | AY090140 |

**Supplementary Table S2.** Taxon sampling (*new sampled specimen) in species-level tree with GenBank accession number and main distribution.

| **Number** | **Subfamily/Tribe/Genus** | **Species** | **Genbank accession number** | | | **Main distribution** |
| --- | --- | --- | --- | --- | --- | --- |
|  |  |  | ***COI*** | ***EF1-a*** | ***wingless*** |  |
|  | **Nymphalinae** |  |  |  |  |  |
|  | **Nymphalini** |  |  |  |  |  |
| 1 | *Aglais* | *Aglais urticae** | KX824675 | KX824707 | KX824739 | Palaearctic |
| 2 |  | *Aglais milberti* | AY248787 | AY248812 | AY248828 | Nearctic |
| 3 |  | *Aglais io** | KX824672 | KX824704 | KX824736 | Palaearctic |
| 4 | *Vanessa* | *Vanessa indica** | KX824667 | KX824699 | KX824731 | Palaearctic, Oriental |
| 5 |  | *Vanessa cardui** | KX824666 | KX824698 | KX824730 | Nearctic, Palaearctic, Oriental, Australasian and Afrotropical |
| 6 |  | *Vanessa annabella* | AY788685 | AY788823 | AY788583 | Nearctic |
| 7 |  | *Vanessa virginiensis* | AY248783 | AY248808 | AY248827 | Nearctic |
| 8 |  | *Vanessa atalanta* | AY090221 | AY090187 | AF412772 | Nearctic, Palaearctic and Afrotropical |
| 9 |  | *Vanessa braziliensis* | AY788686 | AY788824 | AY788584 | Neotropical |
| 10 |  | *Vanessa gonerilla* | AY248784 | AY248809 | AF412782 | Australasian |
| 11 |  | *Vanessa kershawi* | AY788689 | AY788827 | AY788587 | Australasian |
| 12 |  | *Vanessa myrinna* | AY788690 | AY788828 | AY788588 | Neotropical |
| 13 | *Polygonia* | *Polygonia c-album** | KX824664 | KX824696 | KX824728 | Palaearctic, Afrotropical |
| 14 |  | *Polygonia c-aureun** | KX824665 | KX824697 | KX824729 | Palaearctic |
| 15 |  | *Polygonia comma* | AY248794 | AY248819 | AF412781 | Nearctic |
| 16 |  | *Polygonia faunus* | AY248798 | AY248823 | AY248837 | Nearctic |
| 17 |  | *Polygonia egea* | AY248800 | AY248825 | AY248838 | Palaearctic |
| 18 |  | *Polygonia haroldi* | AY788662 | AY788800 | AY788560 | Nearctic |
| 19 | *Kaniska* | *Kaniska canace** | KX824674 | KX824706 | KX824738 | Oriental |
| 20 | *Nymphalis* | *Nymphalis antiopa** | KX824668 | KX824700 | KX824732 | Nearctic, Palaearctic and Neotropical |
| 21 |  | *Nymphalis xanthomelas** | KX824670 | KX824702 | KX824734 | Palaearctic, Oriental |
| 22 |  | *Nymphalis vau-album** | KX824669 | KX824701 | KX824733 | Nearctic, Palaearctic |
| 23 |  | *Nymphalis polychloros* | AY248788 | AY248813 | AY248829 | Palaearctic, Afrotropical |
| 24 |  | *Nymphalis californica* | AY248789 | AY248814 | AY248830 | Nearctic |
| 25 | *Symbrenthia* | *Symbrenthia niphanda** | KX824679 | KX824711 | KX824743 | Oriental |
| 26 |  | *Symbrenthia hypselis** | KX824677 | KX824709 | KX824741 | Oriental |
| 27 |  | *Symbrenthia brabira** | KX824676 | KX824708 | KX824740 | Oriental |
| 28 |  | *Symbrenthia lilea** | KX824678 | KX824710 | KX824742 | Oriental |
| 29 |  | *Symbrenthia hypatia* | AY248779 | AY248804 | AF412784 | Australasian |
| 30 | *Araschnia* | *Araschnia prorsoides** | KX824692 | KX824724 | KX824756 | Palaearctic |
| 31 |  | *Araschnia doris** | KX824691 | KX824723 | KX824755 | Oriental |
| 32 |  | *Araschnia burejana** | KX824689 | KX824721 | KX824753 | Oriental |
| 33 |  | *Araschnia dohertyi ** | KX824690 | KX824722 | KX824754 | Palaearctic |
| 34 |  | *Araschnia levana* | AY248780 | AY248805 | AF412762 | Palaearctic |
| 35 | *Antanartia* | *Antanartia delius* | AY788610 | AY788712 | AY788473 | Afrotropical |
| 36 |  | *Antanartia schaenia* | AY218236 | AY218255 | AF412780 | Afrotropical |
| 37 | *Hypanartia* | *Hypanartia bella* | AY788638 | AY788757 | AF246590 | Neotropical |
| 38 |  | *Hypanartia charon* | AY788639 | AY788758 | AY788518 | Neotropical |
| 39 |  | *Hypanartia kefersteini* | AY788640 | AY788759 | AY788519 | Neotropical |
| 40 |  | *Hypanartia lethe* | AF187774 | AY788760 | AY788520 | Neotropical, Nearctic |
| 41 | *Colobura* | *Colobura dirce* | AY090228 | AY090196 | AY090162 | Neotropical |
| 42 | *Smyrna* | *Smyrna blomfildia* | AY788678 | AY788816 | AY788576 | Neotropical, Nearctic |
| 43 | *Mynes* | *Mynes geoffroyi* | AY248778 | AY248803 | AF412760 | Australasian |
| 44 | *Tigridia* | *Tigridia acesta* | AY788684 | AY788822 | AY788582 | Neotropical |
|  | **Melitaeini** |  |  |  |  |  |
| 45 | *Euphydryas* | *Euphydryas intermedia** | KX824671 | KX824703 | KX824735 | Palaearctic |
| 46 |  | *Euphydryas aurinia* | AF187746 | AY788743 | AY788504 | Palaearctic |
| 47 |  | *Euphydryas desfontainii* | AY090226 | AY090193 | AY090159 | Palaearctic |
| 48 |  | *Euphydryas chalcedona* | AF187752 | AY788744 | AY788505 | Nearctic |
| 49 |  | *Euphydryas gillettii* | AF187771 | AY788746 | AY788507 | Nearctic |
| 50 |  | *Euphydryas phaeton* | AF187797 | AY788747 | AY788508 | Nearctic |
| 51 | *Chlosyne* | *Chlosyne acastus* | AF187735 | AY788725 | AY788486 | Nearctic |
| 52 |  | *Chlosyne cyneas* | AF187757 | AY788726 | AY788487 | Nearctic |
| 53 |  | *Chlosyne harrisii* | AF187773 | AY788729 | AY788490 | Nearctic |
| 54 |  | *Chlosyne palla* | AF187791 | AY788733 | AY788494 | Nearctic |
| 55 |  | *Chlosyne narva* | AF187786 | AY788731 | AY788492 | Neotropical, Nearctic |
| 56 | *Melitaea* | *Melitaea didymoides** | KX824681 | KX824713 | KX824745 | Palaearctic |
| 57 |  | *Melitaea diamina** | KX824680 | KX824712 | KX824744 | Palaearctic, Afrotropical |
| 58 |  | *Melitaea protomedia** | KX824682 | KX824714 | KX824746 | Palaearctic, Oriental |
| 59 |  | *Melitaea scotosia* | AF187804 | AY788780 | AY788540 | Palaearctic |
| 60 |  | *Melitaea arduinna* | AF187742 | AY788774 | AY788534 | Palaearctic |
| 61 |  | *Melitaea cinxia* | AY788656 | AY788776 | AY788536 | Palaearctic, Afrotropical |
| 62 |  | *Melitaea latonigena* | AF187780 | AY788778 | AY788538 | Palaearctic |
| 63 |  | *Melitaea persea* | AF187796 | AY788779 | AY788539 | Oriental, Afrotropical |
| 64 |  | *Melitaea deione* | AY788657 | AY788777 | AY788537 | Palaearctic |
| 65 | *Phyciodes* | *Phyciodes batesii* | AF187747 | AY788789 | AY788549 | Nearctic |
| 66 |  | *Phyciodes orseis* | AY156631 | AY788792 | AY788552 | Nearctic |
| 67 |  | *Phyciodes pallida* | AF187792 | AY788794 | AY788554 | Nearctic |
| 68 |  | *Phyciodes pulchella* | AY156662 | AY788797 | AY788557 | Nearctic |
| 69 |  | *Phyciodes tharos* | AF187807 | AY788798 | AY788558 | Nearctic |
| 70 |  | *Phyciodes pallescens* | AY156640 | AY788793 | AY788553 | Nearctic |
| 71 |  | *Phyciodes phaon* | AF187798 | AY788795 | AY788555 | Nearctic |
| 72 |  | *Phyciodes graphica* | AY156684 | AY788790 | AY788550 | Nearctic |
| 73 | *Poladryas* | *Poladryas arachne* | AF187740 | AY788799 | AY788559 | Neotropical, Nearctic |
| 74 | *Microtia* | *Microtia elva* | AY788660 | AY788787 | AY788547 | Neotropical, Nearctic |
| 75 | *Gnathotriche* | *Gnathotriche exclamationis* | AY788629 | AY788748 | AY788509 | Neotropical |
| 76 | *Higginsius* | *Higginsius fasciatus* | AY788630 | AY788749 | AY788510 | Neotropical |
| 77 | *Antillea* | *Antillea pelops* | GQ864733 | GQ864827 | GQ864421 | Neotropical |
| 78 |  | *Antillea proclea* | EF493928 | EF493976 | EF493869 | Neotropical |
| 79 | *Atlantea* | *Atlantea pantoni* | GQ864741 | GQ864835 | GQ864429 | Neotropical |
| 80 | *Phystis* | *Phystis simois* | EF493956 | EF494014 | EF493907 | Neotropical |
| 81 | *Mazia* | *Mazia amazonica* | AY788654 | AY788773 | AY788533 | Neotropical |
| 82 | *Ortilia* | *Ortilia gentina* | EF493950 | EF494000 | EF493893 | Neotropical |
| 83 |  | *Ortilia liriope* | EF493972 | EF494031 | EF493924 | Neotropical |
| 84 |  | *Ortilia orticas* | EF493952 | EF494002 | EF493895 | Neotropical |
| 85 | *Tegosa* | *Tegosa anieta* | AY788681 | AY788819 | AY788579 | Neotropical, Nearctic |
| 86 |  | *Tegosa tissoides* | AY788682 | AY788820 | AY788580 | Neotropical |
| 87 | *Texola* | *Texola elada* | AY788659 | AY788786 | AY788546 | Nearctic |
| 88 | *Anthanassa* | *Anthanassa drusilla* | AY788611 | AY788714 | AY788475 | Neotropical, Nearctic |
| 89 |  | *Anthanassa texana* | AF187806 | AY788716 | AY788477 | Neotropical, Nearctic |
| 90 |  | *Anthanassa ardys* | AF187743 | AY788713 | AY788474 | Neotropical, Nearctic |
| 91 |  | *Anthanassa tulcis* | AY788612 | AY788717 | AY788478 | Neotropical, Nearctic |
| 92 | *Castilia* | *Castilia eranites* | AY788617 | AY788722 | AY788483 | Neotropical |
| 93 |  | *Castilia ofella* | AY788618 | AY788723 | AY788484 | Neotropical |
| 94 | *Telenassa* | *Telenassa trimaculata* | AY788683 | AY788821 | AY788581 | Neotropical |
| 95 | *Dagon* | *Dagon pusillus* | EF493932 | EF493982 | EF493875 | Neotropical |
| 96 | *Janatella* | *Janatella leucodesma* | AY788641 | AY788761 | AY788521 | Neotropical |
| 97 | *Eresia* | *Eresia clio* | AY788622 | AY788736 | AY788497 | Neotropical |
| 98 |  | *Eresia coela* | AY788623 | AY788737 | AY788498 | Neotropical |
| 99 |  | *Eresia eunice* | AY788624 | AY788738 | AY788499 | Neotropical |
| 100 |  | *Eresia pelonia* | AY788626 | AY788740 | AY788501 | Neotropical |
| 101 | *Dymasia* | *Dymasia dymas* | AF187764 | AY788785 | AY788545 | Nearctic |
|  | **Kallimini** |  |  |  |  |  |
| 102 | *Doleschallia* | *Doleschallia bisaltide** | KX824663 | KX824695 | KX824727 | Oriental, Australasian |
| 103 | *Kallima* | *Kallima inachus** | KX824673 | KX824705 | KX824737 | Oriental |
| 104 |  | *Kallima paralekta* | AY090229 | AY090197 | AY090163 | Oriental |
| 105 | *Catacroptera* | *Catacroptera cloanthe* | AY788619 | AY788724 | AY788485 | Afrotropical |
| 106 | *Mallika* | *Mallika jacksoni* | AY788653 | AY788772 | AY788532 | Afrotropical |
|  | **Junoniini** |  |  |  |  |  |
| 107 | *Hypolimnas* | *Hypolimnas missipus** | KX824662 | KX824694 | KX824726 | Oriental |
| 108 |  | *Hypolimnas bolina** | KX824661 | KX824693 | KX824725 | Oriental |
| 109 |  | *Hypolimnas alimena* | AY788633 | AY788752 | AY788513 | Australasian |
| 110 |  | *Hypolimnas anthedon* | AY788634 | AY788753 | AY788514 | Afrotropical |
| 111 |  | *Hypolimnas pandarus* | AY788636 | AY788755 | AY788516 | Oriental |
| 112 | *Junonia* | *Junonia almana** | KX824683 | KX824715 | KX824747 | Oriental |
| 113 |  | *Junonia hierta** | KX824685 | KX824717 | KX824749 | Oriental, Afrotropical |
| 114 |  | *Junonia lemonias** | KX824687 | KX824719 | KX824751 | Oriental |
| 115 |  | *Junonia iphita** | KX824686 | KX824718 | KX824750 | Oriental |
| 116 |  | *Junonia atlites** | KX824684 | KX824716 | KX824748 | Oriental |
| 117 |  | *Junonia touhilimasa* | AY788649 | AY788768 | AY788528 | Afrotropical |
| 118 |  | *Junonia coenia* | AY788643 | AY248801 | AY248826 | Nearctic |
| 119 |  | *Junonia erigone* | AY788644 | AY788763 | AY788523 | Oriental, Australasian |
| 120 |  | *Junonia natalica* | AY788645 | AY788764 | AY788524 | Afrotropical |
| 121 |  | *Junonia sophia* | AY788647 | AY788766 | AY788526 | Afrotropical |
| 122 | *Yoma* | *Yoma sabina** | KX824688 | KX824720 | KX824752 | Oriental, Australasian |
| 123 |  | *Yoma algina* | AY788692 | AY788830 | AY788590 | Australasian |
| 124 | *Salamis* | *Salamis anteva* | AY788675 | AY788813 | AY788573 | Afrotropical |
| 125 |  | *Salamis cacta* | AY788676 | AY788814 | AY788574 | Afrotropical |
| 126 | *Protogoniomorpha* | *Protogoniomorpha anacardii* | AY090223 | AY090189 | AY090155 | Afrotropical |
| 127 |  | *Protogoniomorpha parhassus* | AY788673 | AY788811 | AY788571 | Afrotropical |
| 128 | *Precis* | *Precis andremiaja* | AY788664 | AY788802 | AY788562 | Afrotropical |
| 129 |  | *Precis cuama* | AY788668 | AY788806 | AY788566 | Afrotropical |
| 130 |  | *Precis octavia* | AY788669 | AY788807 | AY788567 | Afrotropical |
| 131 |  | *Precis sinuata* | AY788670 | AY788808 | AY788568 | Afrotropical |
| 132 |  | *Precis tugela* | AY788671 | AY788809 | AY788569 | Afrotropical |
|  | **Victorinini** |  |  |  |  |  |
| 133 | *Rhinopalpa* | *Rhinopalpa polynice* | AY788674 | AY788812 | AY788572 | Oriental |
| 134 | *Kallimoides* | *Kallimoides rumia* | AY788651 | AY788770 | AY788530 | Afrotropical |
| 135 | *Vanessula* | *Vanessula milca* | AY788691 | AY788829 | AY788589 | Afrotropical |
| 136 | *Siproeta* | *Siproeta epaphus* | AY788677 | AY788815 | AY788575 | Neotropical, Nearctic |
| 137 | *Napeocles* | *Napeocles jucunda* | AY788661 | AY788788 | AY788548 | Neotropical |
| 138 | *Anartia* | *Anartia fatima* | AY788607 | AY788709 | AY788470 | Nearctic |
| 139 |  | *Anartia jatrophae* | AY788608 | AY788710 | AY788471 | Neotropical, Nearctic |
| 140 | *Metamorpha* | *Metamorpha elissa* | AY788658 | AY788784 | AY788544 | Neotropical |
|  | **Coeini** |  |  |  |  |  |
| 141 | *Pycina* | *Pycina zamba* | GQ864804 | GQ864898 | GQ864492 | Neotropical, Nearctic |
| 142 | *Baeotus* | *Baeotus deucalion* | AY788616 | AY788721 | AY788482 | Neotropical |
| 143 | *Historis* | *Historis odius* | AY788632 | AY788751 | AY788512 | Neotropical, Nearctic |
| 144 |  | *Historis acheronta* | AY788631 | AY788750 | AY788511 | Neotropical, Nearctic |
|  | **Outgroup** |  |  |  |  |  |
|  | **Cyrestinae** |  |  |  |  |  |
| 145 | *Cyrestis* | *Cyrestis thyodamas* | AY218240 | AY218260 | AY218278 | Oriental |
|  | **Heliconiinae** |  |  |  |  |  |
| 146 | *Heliconius* | *Heliconius hecale* | AY090202 | AY090168 | AY090135 | Neotropical, Nearctic |
|  | **Limenitidinae** |  |  |  |  |  |
| 147 | *Adelpha* | *Adelpha bredowii* | AY788591 | AY788693 | AY788457 | Neotropical, Nearctic |
|  | **Pseudergolinae** |  |  |  |  |  |
| 148 | *Amnosia* | *Amnosia decora* | AY218235 | AY218254 | AY218273 | Oriental |
| 149 | *Dichorragia* | *Dichorragia nesimachus* | AY788602 | AY788704 | AY788466 | Oriental |
| 150 | *Pseudergolis* | *Pseudergolis wedah* | AY788605 | AY788707 | AY788468 | Oriental |
|  | **Biblidinae** |  |  |  |  |  |
| 151 | *Ariadne* | *Ariadne enotrea* | AY218237 | AY218256 | AY218274 | Afrotropical |
| 152 | *Byblia* | *Byblia anvatara* | AY788595 | AY788697 | AY788460 | Afrotropical |
|  | **Apaturinae** |  |  |  |  |  |
| 153 | *Asterocampa* | *Asterocampa leilia* | AF187734 | AY218257 | AY218275 | Nearctic |
| 154 | *Eulaceura* | *Eulaceura osteria* | AY788593 | AY788695 | AF246588 | Oriental |
| 155 | *Mimathyma* | *Mimathyma schrenckii* | AY788594 | AY788696 | AY788459 | Palaearctic |
|  | **Libytheinae** |  |  |  |  |  |
| 156 | *Libytheana* | *Libytheana carinenta* | GQ864786 | GQ864880 | GQ864474 | Neotropical, Nearctic |
| 157 | *Libytheana* | *Libytheana terena* | EU141364 | EU136671 | EU141245 | Neotropical |
| 158 | *Libythea* | *Libythea myrrha* | KT286524 | KT286217 | KT286044 | Oriental |
|  | **Danainae** |  |  |  |  |  |
| 159 | *Danaus* | *Danaus chrysippus* | KP007638 | JX185963 | KP007893 | Oriental, Australasian and Afrotropical |
| 160 | *Danaus* | *Danaus plexippus* | DQ018954 | DQ018921 | AF246564 | Neotropical, Nearctic and Australasian |
| 161 | *Euploea* | *Euploea mulciber* | KM102678 | GU365940 | GU365959 | Oriental |
|  | **Satyrinae** |  |  |  |  |  |
| 162 | *Eumenis* | *Eumenis autonoe* | GU372518 | GU372609 | KM200294 | Palaearctic |
| 163 | *Melanitis* | *Melanitis leda* | AY090207 | AY090173 | AY090140 | Oriental, Australasian and Afrotropical |

**Supplementary Table S3.** Substitution model of each partition with different partitioning schemes.

| Scheme | Total Lenth | Substitution Model (AICc) |
| --- | --- | --- |
| unpartitioned | COI+EF-1α+Wg | GTR+I+G |
| partitioned into mitochondrial and nuclear gene | COI | GTR+I+G |
|  | EF-1α+Wg | GTR+I+G |
| partitioned into individual gene | COI | GTR+I+G |
|  | EF-1α | GTR+I+G |
|  | Wg | K80+G |
| partitioned by PartitionFinder with greedy search scheme (unlinked) | COI coden position 1 and 2 + EF-1α+Wg | GTR+I+G |
|  | COI coden position 3 | GTR+G |
| partitioned by PartitionFinder with user-specified search scheme (unlinked) | COI | GTR+I+G |
|  | EF-1α+Wg | GTR+I+G |

**Supplementary Table S4.** Comparison of crown ages of major lineages and the most recent common ancestor (MRCA) of major splits in Nymphalinae under the modified Calibration Plan 1A (using the same maximum constraints, but with the different minimum time constraints for coevolved butterfly clades, see under Methods). In the Calibration Plan 1A-M5, 1A-M15 and 1A-M20, the minimum time constraints derived by the maximum minus 5, 15 and 20 million years, respectively. Notes: Numbers in brackets refer to 95% HPD intervals of clade ages (note that because HPD intervals are calculated from all trees, node ages occasionally fall outside the interval).

| **Major lineage/split** | **Node** | **Calibration Plans** | | | | | | |
| --- | --- | --- | --- | --- | --- | --- | --- | --- |
|  |  | **1A** |  | **1A-M5** |  | **1A-M15** |  | **1A-M20** |
| Nymphalinae | 1 | 85.1[73.9-98.2] |  | 88.4[78.8-101.6] |  | 81.1[68.7-95.4] |  | 78.6[64.8-92.6] |
| Nymphalini/Kallimoid clade | 2 | 78.7[72.3-85.4] |  | 82.1[77.0-87.3] |  | 74.9[66.6-82.9] |  | 72.4[63.4-82.0] |
| Nymphalini | 3 | 59.7[50.7-69.3] |  | 60.9[51.6-71.3] |  | 57.8[48.6-67.0] |  | 56.6[47.7-66.2] |
| Kallimoid clade | 4 | 74.9[70.4-79.7] |  | 78.4[75.6-81.1] |  | 70.8[64.1-77.7] |  | 68.4[60.5-76.8] |
| Old Nymphalini | 5 | 45.4[39.5-51.3] |  | 46.0[40.4-52.4] |  | 44.5[38.9-50.3] |  | 43.8[38.6-49.8] |
| Hypanartia/sister clade | 6 | 42.9[37.1-49.1] |  | 43.5[37.4-49.6] |  | 42.0[36.3-48.2] |  | 41.3[35.7-47.4] |
| Hypanartia | 7 | 29.2[21.7-36.5] |  | 29.6[22.1-37.0] |  | 28.6[21.5-36.2] |  | 28.0[20.6-35.1] |
| Vanessa/Nymphalis-group | 8 | 35.9[34.0-38.1] |  | 36.0[33.9-38.0] |  | 35.8[33.8-37.8] |  | 35.7[33.7-37.7] |
| Vanessa | 9 | 26.4[21.0-31.9] |  | 26.6[21.2-31.8] |  | 26.1[20.9-31.7] |  | 25.7[19.8-31.0] |
| Nymphalis-group | 10 | 28.9[24.3-33.1] |  | 29.1[24.7-33.4] |  | 28.6[24.1-33.1] |  | 28.3[23.5-33.0] |
| Victorinini+Junoniini/Kallimini | 11 | 64.8[55.3-74.4] |  | 67.0[57.4-77.1] |  | 62.0[52.6-72.0] |  | 60.3[50.8-70.5] |
| Kallimini | 12 | 40.2[27.6-53.9] |  | 41.4[28.1-56.3] |  | 38.9[26.9-52.0] |  | 37.9[25.9-50.3] |
| Victorinini/Junoniini | 13 | 56.2[47.3-64.5] |  | 57.7[48.8-66.6] |  | 54.1[44.8-62.2] |  | 52.6[44.4-61.9] |
| Victorinini | 14 | 50.5[42.1-59.0] |  | 51.9[43.2-60.1] |  | 48.7[40.3-57.5] |  | 47.3[38.9-56.4] |
| Junoniini | 15 | 42.3[35.3-49.8] |  | 43.1[35.7-50.5] |  | 41.0[33.9-48.4] |  | 39.9[32.7-47.5] |
| Junonia/sister clade | 16 | 36.5[29.9-43.3] |  | 37.2[30.8-44.7] |  | 35.5[28.8-42.4] |  | 34.6[28.1-42.0] |
| Junonia | 17 | 24.7[19.3-30.1] |  | 25.1[19.6-30.4] |  | 24.1[18.8-29.8] |  | 23.5[18.5-29.3] |
| Melitaeini/sister clade | 18 | 68.5[62.5-74.7] |  | 71.0[64.6-76.4] |  | 65.5[58.7-72.9] |  | 63.4[55.4-71.9] |
| Melitaeini | 19 | 56.6[50.5-62.7] |  | 57.4[51.7-63.8] |  | 55.1[48.2-61.8] |  | 53.8[46.3-61.2] |
| Chlosyne-group/sister clade | 20 | 47.8[43.5-51.9] |  | 47.9[45.1-50.6] |  | 47.2[41.7-52.9] |  | 46.3[40.2-52.8] |
| Chlosyne-group | 21 | 41.7[35.0-48.0] |  | 41.9[35.8-47.7] |  | 41.1[33.9-48.3] |  | 40.2[32.7-47.9] |
| Melitaea/sister clade | 22 | 39.5[33.4-44.8] |  | 39.8[34.5-44.7] |  | 39.0[32.5-45.2] |  | 38.2[31.6-45.1] |
| Melitaea | 23 | 32.1[26.0-38.1] |  | 32.4[26.7-38.5] |  | 31.5[24.6-37.6] |  | 30.9[24.3-37.8] |

**Supplementary Table S5.** Dispersal rate scaling matrices used in Lagrange analyses under Dispersal-Extinction-Cladogenesis (DEC) model and Statistical DEC (Bays-Lagrange, S-DEC) model. The five time slices are shown, providing the connectivity between the biogeographical areas along the time. A, Neotropical; B, Nearctic; C, Palaearctic; D, Oriental; E, Australasian; F, Afrotropical (see Fig. 2a).

| **21-0 Ma** |  |  |  |  |  |  |
| --- | --- | --- | --- | --- | --- | --- |
|  | **A** | **B** | **C** | **D** | **E** | **F** |
| **A0-1** | 1 | 1 | 0.5 | 0.5 | 0.01 | 0.01 |
| **B0-1** | 1 | 1 | 1 | 1 | 0.01 | 0.5 |
| **C0-1** | 0.5 | 1 | 1 | 1 | 0.5 | 1 |
| **D0-1** | 0.5 | 1 | 1 | 1 | 0.5 | 1 |
| **E0-1** | 0.01 | 0.01 | 0.5 | 0.5 | 1 | 0.5 |
| **F0-1** | 0.01 | 0.5 | 1 | 1 | 0.5 | 1 |
|  |  |  |  |  |  |  |
| **35-21 Ma** |  |  |  |  |  |  |
|  | **A** | **B** | **C** | **D** | **E** | **F** |
| **A1-2** | 1 | 1 | 0.5 | 0.5 | 0.01 | 0.01 |
| **B1-2** | 1 | 1 | 1 | 1 | 0.01 | 0.5 |
| **C1-2** | 0.5 | 1 | 1 | 1 | 0.5 | 1 |
| **D1-2** | 0.5 | 1 | 1 | 1 | 0.5 | 0.5 |
| **E1-2** | 0.01 | 0.01 | 0.5 | 0.5 | 1 | 0.01 |
| **F1-2** | 0.01 | 0.5 | 1 | 0.5 | 0.01 | 1 |
|  |  |  |  |  |  |  |
| **56-35 Ma** |  |  |  |  |  |  |
|  | **A** | **B** | **C** | **D** | **E** | **F** |
| **A2-3** | 1 | 0.01 | 0.01 | 0.01 | 0.01 | 0.01 |
| **B2-3** | 0.01 | 1 | 1 | 1 | 0.01 | 0.5 |
| **C2-3** | 0.01 | 1 | 1 | 1 | 0.01 | 1 |
| **D2-3** | 0.01 | 1 | 1 | 1 | 0.01 | 0.5 |
| **E2-3** | 0.01 | 0.01 | 0.01 | 0.01 | 1 | 0.01 |
| **F2-3** | 0.01 | 0.5 | 1 | 0.5 | 0.01 | 1 |
|  |  |  |  |  |  |  |
| **60-56 Ma** |  |  |  |  |  |  |
|  | **A** | **B** | **C** | **D** | **E** | **F** |
| **A3-4** | 1 | 0.01 | 0.01 | 0.01 | 0.01 | 0.5 |
| **B3-4** | 0.01 | 1 | 1 | 1 | 0.01 | 0.5 |
| **C3-4** | 0.01 | 1 | 1 | 1 | 0.01 | 1 |
| **D3-4** | 0.01 | 1 | 1 | 1 | 0.01 | 0.5 |
| **E3-4** | 0.01 | 0.01 | 0.01 | 0.01 | 1 | 0.01 |
| **F3-4** | 0.5 | 0.5 | 1 | 0.5 | 0.01 | 1 |
|  |  |  |  |  |  |  |
| **85-60 Ma** |  |  |  |  |  |  |
|  | **A** | **B** | **C** | **D** | **E** | **F** |
| **A4-5** | 1 | 0.01 | 0.01 | 0.01 | 0.5 | 0.5 |
| **B4-5** | 0.01 | 1 | 1 | 1 | 0.01 | 0.01 |
| **C4-5** | 0.01 | 1 | 1 | 1 | 0.01 | 0.01 |
| **D4-5** | 0.01 | 1 | 1 | 1 | 0.01 | 0.01 |
| **E4-5** | 0.5 | 0.01 | 0.01 | 0.01 | 1 | 0.01 |
| **F4-5** | 0.5 | 0.01 | 0.01 | 0.01 | 0.01 | 1 |

**Supplementary Figure S1.** The species-level phylogenetic relationships of Nymphalinae based on maximum likelihood and Bayesian analyses (see under Methods).


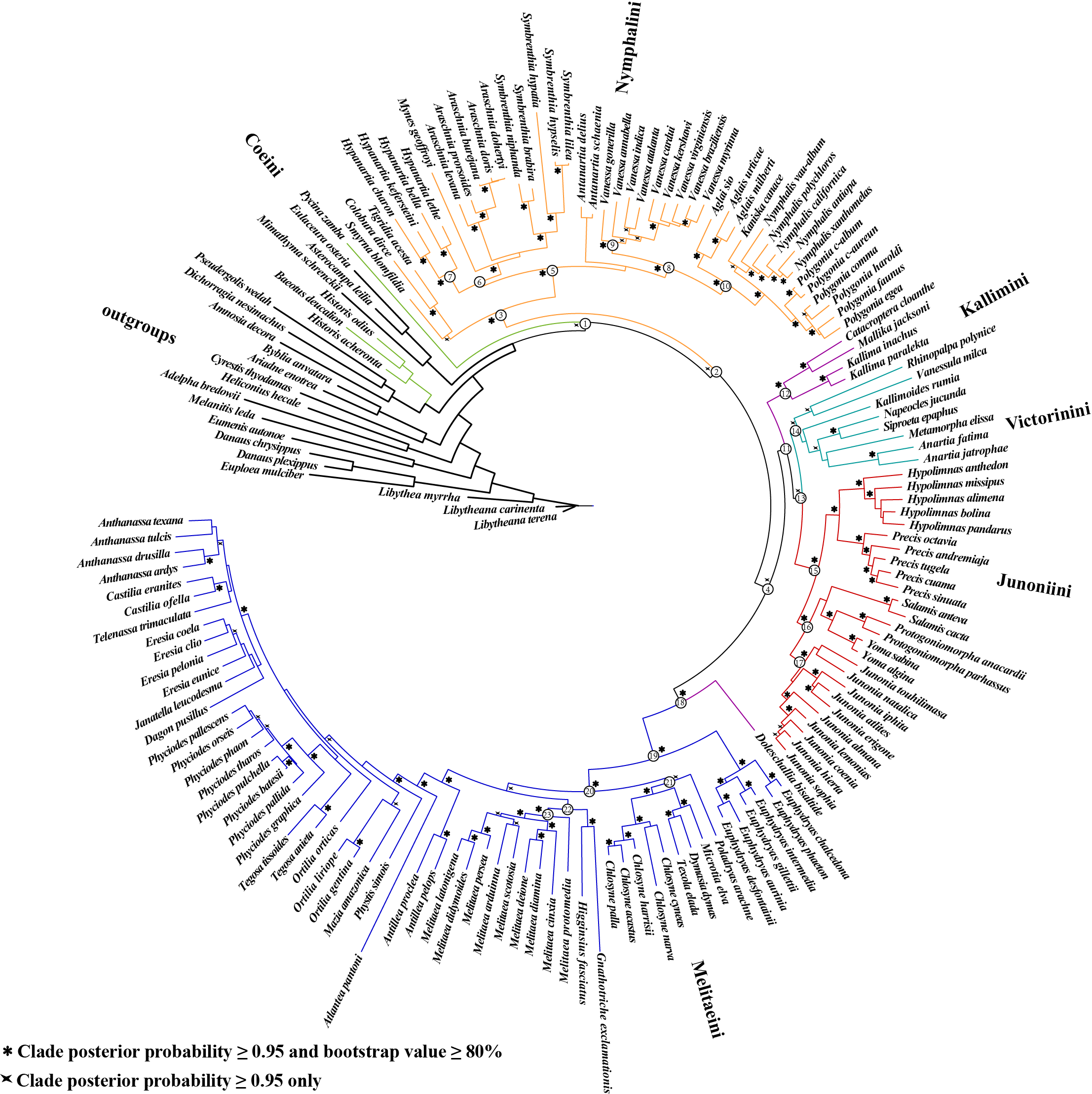


**Supplementary Figure S2.** Inter-continental routes of dispersal for major clades of Nymphalinae according to Table 2. Star (★) represents the probable center of early divergences for Nymphalinae after the Cretaceous-Paleogene mass extinction. The map of biogeographic realms freely from https://commons.wikimedia.org/wiki/File:Ecozones.svg following the same license terms (https://creativecommons.org/licenses/by-sa/4.0/legalcode), and drawn with Adobe Illustrator CS5 by Chengyong Su.


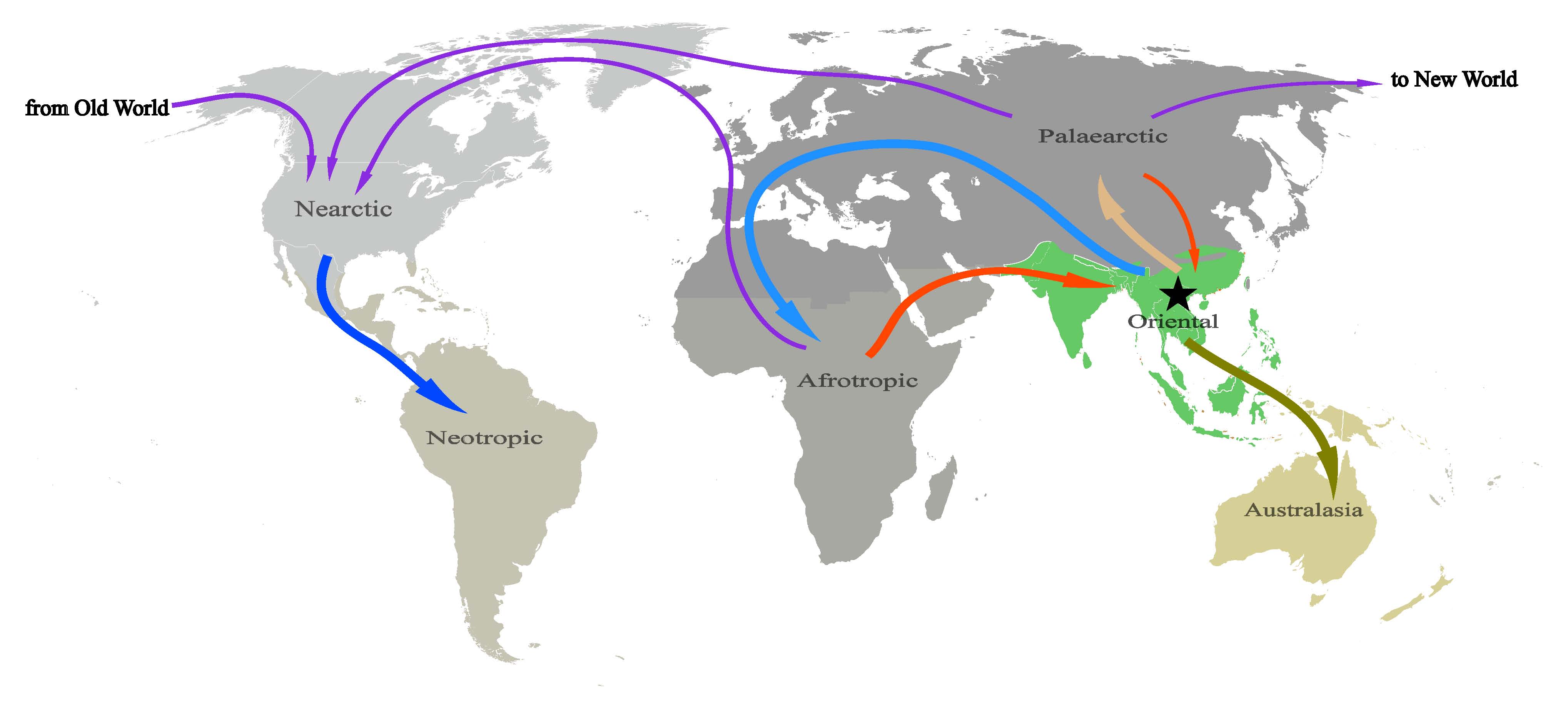

Supplement: Supplementary file 1 — Supplementary Information [file 41598_2017_8993_MOESM1_ESM.doc]
